# Identification of reference genes for RT-qPCR in the Antarctic moss *Sanionia uncinata* under abiotic stress conditions

**DOI:** 10.1371/journal.pone.0199356

**Published:** 2018-06-19

**Authors:** Mira Park, Soon Gyu Hong, Hyun Park, Byeong-ha Lee, Hyoungseok Lee

**Affiliations:** 1 Unit of Polar Genomics, Korea Polar Research Institute, Incheon, South Korea; 2 Department of Life Sciences, Sogang University, Seoul, South Korea; 3 Division of Life Sciences, Korea Polar Research Institute, Incheon, South Korea; 4 Polar Science, University of Science & Technology, Daejeon, South Korea; University of Parma, ITALY

## Abstract

*Sanionia uncinata* is a dominant moss species in the maritime Antarctic. Due to its high adaptability to harsh environments, this extremophile plant has been considered a good target for studying the molecular adaptation mechanisms of plants to a variety of environmental stresses. Despite the importance of *S*. *uncinata* as a representative Antarctic plant species for the identification and characterization of genes associated with abiotic stress tolerance, suitable reference genes, which are critical for RT-qPCR analyses, have not yet been identified. In this report, 11 traditionally used and 6 novel candidate reference genes were selected from transcriptome data of *S*. *uncinata* and the expression stability of these genes was evaluated under various abiotic stress conditions using three statistical algorithms; geNorm, NormFinder, and BestKeeper. The stability ranking analysis selected the best reference genes depending on the stress conditions. Among the 17 candidates, the most stable references were *POB1* and *UFD2* for cold stress, *POB1* and *AKB* for drought treatment, and *UFD2* and *AKB* for the field samples from a different water contents in Antarctica. Overall, novel genes *POB1* and *AKB* were the most reliable references across all samples, irrespective of experimental conditions. In addition, 6 novel candidate genes including *AKB*, *POB1* and *UFD2*, were more stable than the housekeeping genes traditionally used for internal controls, indicating that transcriptome data can be useful for identifying novel robust normalizers. The reference genes validated in this study will be useful for improving the accuracy of RT-qPCR analysis for gene expression studies of *S*. *uncinata* in Antarctica and for further functional genomic analysis of bryophytes.

## Introduction

Bryophytes dominate polar regions by developing adaptive strategies, as reflected in their poikilohydric lifestyle, to withstand an array of harsh conditions [1]. In extremely cold and/or dry conditions, temperature and water availability are the most influential factors with respect to plant growth and carbon gain, which determine bryophyte productivity in the Antarctic [2, 3]. These plants have evolved desiccation tolerance by rapidly recovering net positive photosynthesis within minutes of rehydration [1].

The moss *Sanionia uncinata* (Hedw.) Loeske is one of the most dominant species of moss in both the Arctic and Antarctic regions and is widely distributed over coastal areas [4, 5]. This pleurocarpous moss species forms carpets and occurs in a wide variety of habitats, ranging from dry sites such as exposed rock surfaces to wet sites near streams and swampy ground [6–8]. It has recently been reported that *S*. *uncinata* is more tolerant to desiccation stress than *Polytrichastrum alpinum*. *S*. *uncinata* showed a more rapid rate of recovery from complete water loss and could retain water in its tissues for a longer period of time compared with *P*. *alpinum* [9]. However, the molecular mechanisms underlying stress tolerance have not yet been identified. Understanding the expression patterns of stress response genes can help discover the tolerance mechanisms of *S*. *uncinata* in response to various abiotic stresses in extreme environments.

Gene expression analysis is essential for uncovering the molecular mechanisms underlying various stress tolerances. Reverse transcription quantitative real-time polymerase chain reaction (RT-qPCR) is one of the most widely used techniques for gene expression studies due to its high sensitivity and specificity, with high-throughput capabilities and cost-effectiveness [10, 11]. However, when conducting gene expression analyses by RT-qPCR, various factors such as RNA quality, reverse transcription efficiency, cDNA quality, and amplification efficiency can significantly influence the reliability of the results [12]. To reduce the influence of these factors, selection of internal reference genes with stable expression levels is critical for obtaining reliable results [13]. Some reports have suggested that suitable reference genes should be selected in a species-specific manner and tested under particular experimental conditions when validating their reliability [14, 15].To ensure more precise results, it is necessary to use one or more stable reference genes to normalize the expression data of target genes [16, 17].

Housekeeping genes, such as β-actin (*ACT*), glyceraldehyde-3-phosphate dehydrogenase (*GAPDH*), α- or β-tubulin (*TUB*), 18S ribosomal RNA (*18S*), and ubiquitin (*UBQ*), have been widely used as reference genes for RT-qPCR in plants [18, 19]. However, recent studies have shown that these commonly used reference genes might not be stably expressed under different experimental conditions [13, 20–23] and under various abiotic stress conditions [13, 20, 21, 24], suggesting that the use of unauthenticated references could greatly affect the quantification of expression levels of a target gene. However, few reference genes have been tested in *Physcomitrella patens* [25] and even in extremely stress-tolerant dessert moss *Syntrichia caninervis* and the model liverwort *Marchantia polymorpha* [26, 27]. Therefore, it is necessary to identify and validate suitable reference genes under different experimental conditions prior to gene expression studies in bryophytes.

In this study, we assessed 17 reference genes (11 traditional and 6 novel ones) from the transcriptome data of the Antarctic moss *S*. *uncinata*, and evaluated their reliabilities as internal controls for RT-qPCR analysis under three different experimental conditions; axenically cultured moss gametophytes under cold or drought treatments, and natural field samples with a water-level gradient in the maritime Antarctic. To select the most suitable reference genes, three different statistical tools, geNorm [12], NormFinder [28], and BestKeeper [29] were used to evaluate the expression stability of the candidate reference genes.

## Material and methods

### Plant materials and stress treatments

Samples of *S*. *uncinata* were collected from a coastal area (62°13’13”S 58°46’18”W) on the Barton Peninsula of King George Island in January 2015. To prepare samples from various water gradients in the field (WGF), *S*. *uncinata* patches of 5 cm x 5 cm containing at least 50 gametophytes were collected from three Antarctic field populations exhibiting a series of water contents. They were designated W68, W48, and W32, representing *S*. *uncinata* gametophytes with water contents of 68%, 48%, and 32%, respectively. Four biological replicates were prepared for each condition. Some of the moss gametophytes were transported to the laboratory at the Korea Polar Research Institute and cultivated axenically on BCDAT solid media [26] in a climate chamber at 15°C with a 16-h light/8-h dark cycle. To prepare the cold experimental set, gametophyte colonies grown at 15°C were transferred to chambers at 2°C and incubated for 1, 3, and 8 h. For the drought experimental set, drought treatment was carried out by transferring colonies grown at 15°C to clean dishes, followed by air-drying at room temperature for 6, 12, and 24 h. For each treatment, four biological replicates were prepared and each replicate contained >20 gametophytes to avoid possible variations among individuals. Immediately after the stress treatments, samples were frozen in liquid nitrogen and stored at –80°C until further use (Table 1).

**Table 1:** Growth and abiotic stress conditions tested on Antarctic moss *S*. *uncinata*.

Experimental set	Tissue	condition	Age of culture	Medium
**Cold**	gametophore	cold control, 15°C	40 days	BCDAT
	gametophore	cold 2°C, 1 h	40 days	BCDAT
	gametophore	cold 2°C, 3 h	40 days	BCDAT
	gametophore	cold 2°C, 8 h	40 days	BCDAT
**Drought**	gametophore	drought 0 h, control	40 days	BCDAT
	gametophore	drought 6 h	40 days	BCDAT
	gametophore	drought 12 h	40 days	BCDAT
	gametophore	drought 24 h	40 days	BCDAT
**Water gradient in the field (WGF)**	gametophore	68% water content (W68)		
	gametophore	48% water content (W48)		
	gametophore	32% water content (W32)		

### Total RNA extraction and cDNA synthesis

Total RNA was isolated using the RNeasy Plant Mini Kit (Qiagen). Genomic DNA contamination was eliminated using RNase-free DNaseI (Qiagen). RNA concentration, purity, and integrity were determined using a NanoDrop ND-2000 spectrophotometer (Thermo Fisher Scientific) and visually assessed via gel electrophoresis (S1 Fig). Each RNA sample (2 μg) was reverse-transcribed using the M-MLV transcriptase enzyme (Enzynomics) according to the manufacturer’s instructions. Reverse transcription was performed at 42°C for 60 min in a final volume of 20 μl, and inactivation of the enzyme was achieved at 72°C for 5 min. All cDNA were stored at –20°C until further use.

### Candidate reference gene selection from transcriptome data

Transcriptome sequencing was carried out using total RNA from the cold experimental set, which comprised gametophyte colonies grown at 15°C, transferred to chambers at 2°C, and incubated for 1, 3, and 8 h. Gametophyte samples grown at 15°C were used as a control. Four different biological replicates were prepared. To construct the sequencing library, 2 μg of total RNA of each sample was used as the input for the TruSeq RNA sample prep kit v2 (Illumina). Following validation and quantification, libraries were applied to a MiSeq Sequencer system (Illumina) using the Illumina MiSeq Reagent Kit v3 (600 cycles). After assembly using the CLC Genomics Workbench v8 module (Qiagen), the transcriptome dataset composed of 21,993 unigenes was employed to analyze the read counts, which were converted to fragments per kilobase per million mapped reads (FPKM values) [30]. To estimate the expression stability of each gene, the following indices of FPKM values, including mean expression value (MV), standard deviation (SD), and coefficient of variation (CV) value, which is obtained by dividing SD by MV, were calculated [31]. The genes with an MV above the half value of the average MV for total unigenes and a CV below 0.3 were considered to be stably expressed. In addition to the 12 stable candidates with low CV, 5 additional candidate genes that showed high similarity to known housekeeping genes were included, which showed CV values above 0.3 from the transcriptome analysis. Finally, 17 candidate reference genes were listed with a transcriptome CV value, gene annotation in the NCBI non-redundant database, and feasibility of primer design (S1 Table).

### Primer design and evaluation of candidate reference genes

Sequences of the 17 candidate reference genes are provided in S1 File and were used to design primers via the PrimerQuest program on the Integrated DNA Technologies website (http://sg.idtdna.com/Primerquest/). The criteria for primer design were as follows: melting temperature (T_m_), 59–65°C; primer length, 18–25 base pairs (bp); GC content, 40–55%; and amplicon length, 100–200 bp. Each primer set returned by the program was tested for homology to other genes using BLASTN. To confirm primer specificity, all primer pairs were initially tested by RT-PCR, and the amplification product for each gene was verified by 2% agarose gel electrophoresis. Amplification efficiency (E) was evaluated using a standard curve generated by RT-qPCR using a 10-fold dilution series (1, 1/10, 1/100, 1/1000, and 1/10,000). Primer specificity was confirmed using melting-curve analysis following RT-qPCR and gel electrophoresis analysis of the unique amplicon. A no template control (NTC) reaction was included in every PCR run for each primer set.

### RT-qPCR

RT-qPCR was performed following the guidelines of the Minimum Information for Publication of Quantitative Real-Time PCR Experiments (MIQE) [32]. Each reaction was performed in 10 μL reactions including 2 μL of a 1:30 dilution of cDNA template, 2 μM of each primer, and 5 μL of SYBR Green (Takara). Amplified signals were monitored continuously with the Rotor Gene 3000 Real-Time Thermal Cycler (Qiagen). The amplification protocol was as follows: 5 min of denaturation and enzyme activation at 95°C, followed by 40 cycles at 95°C for 15 s, 55°C for 20 s, and 72°C for 15 s. Three biological replicates for each sample, and four technical replicates of each biological replicate, were used.

### RT-qPCR data analysis of reference gene stability

Quantification cycle (Cq) values indicating the level of gene expression were determined by RT-qPCR amplification of each candidate reference gene from all RNA samples. The RT-qPCR Cq value of each gene was obtained by calculating the arithmetic mean from four technological and three biological replicates. The correlation coefficients (R values), slopes, and corresponding RT-qPCR efficiency (E) were obtained from standard curves. To rank the reference genes based on gene expression stability under the different experimental conditions, we used three different algorithms: geNorm [12], NormFinder [28], and BestKeeper [29]. The Cq value of each reference gene was converted into an input file according to the respective software manuals. For geNorm and NormFinder analysis, the raw Cq values were converted into relative value using the formula Q = 2^−ΔCq^, in which ΔCq equals to each corresponding Cq minus minimum Cq. These values were then submitted to the Excel-based geNorm applet to calculate the expression stability value (M), which is described as the average of the pairwise variation (V) of a candidate reference gene [12]. Also NormFinder was used to calculate the stability value using an ANOVA-based model to consider intra-group and inter-group variation of the candidate reference genes, with the lowest value representing the highest stability [28]. For BestKeeper, the raw data (Cq values) were used to calculate the coefficients of variance (CV) and the standard deviation (SD), with the lowest CV representing the highest stability [29]. Finally, three results of the stability rankings of 17 candidate reference genes were integrated to determine a comprehensive ranking by calculating the geometric mean of three rankings [33].

### Validation of identified reference genes

Two widely known abiotic stress-related genes, late embryogenesis abundant (*LEA*; (MG020651; forward 5’-TGCTCAATGAGTTTCGTGACTA-3’ and reverse 5’-ATGCAAGACCACCACCGATCATAG-3’) and heat shock protein 70 (*HSP70*; MG721539; forward 5’-AAAGAAGGAGCAGGTGTTCTC-3’ and reverse 5’-TTGCCGAGCAGGTTGTT-3’), were selected as target genes to measure the expression stability of the candidate reference genes under cold, drought, and WGF conditions. The gene expression levels were normalized with the two most stable, as well as the two least stable, candidate reference genes identified from this study.

## Results

### Selection of candidate reference genes

To select the candidate reference genes, we first chose stably expressed genes from the *S*. *uncinata* transcriptome data obtained following treatment at 2°C for 1, 3, and 8 h. After transcriptome analysis with the CLC Genomics Workbench v8 module, we defined stably expressed genes as those fulfilling two conditions: coefficient of variation (CV) below 0.3 and mean expression value (MV) above half of the MV of the total genes. From this dataset of stably expressed genes, we identified 6 housekeeping genes with homology to 60S ribosomal protein L23A (*60S897*), ubiquitin-conjugating enzyme E2 (*E2*.*299*), E3 ubiquitin ligase (*E3*.*528*), elongation factor 1-delta (*EF1*.*278*), histone H3.3 isoform X1 (*HIS*), and translation initiation factor eIF-2B (*tIF*), which are commonly used as reference genes in plants. The six genes were designated as the gene group 1.

Meanwhile, we selected 6 novel genes that have not been widely used as stably expressed genes, homologous to adenylate kinase 4 (*AKB*), histone H3 methyltransferase complex subunit (*HMT*), BTB/POZ domain-containing protein (*POB1*), serine palmitoyltransferase 2 (*SPT*), ubiquitin protein ligase (*UFD2*), and an uncharacterized protein (*UK552*), with stable expression patterns under control and cold stress treatments (S1 Table). These genes were designated as the gene group 2.

Additionally, 5 housekeeping genes that are traditionally used as internal reference genes were selected, which were not stably expressed in *S*. *uncinata* transcriptome data with CV above 0.3: Actin 5 (*ACT5*), actin-related protein 9 (*ARP*), glyceraldehyde-3-phosphate dehydrogenase (*GAPDH*), alpha tubulin (*SuTub*), and gamma tubulin (*TUB250*). These genes were designated as the gene group 3 for using as control to evaluate the efficiency of reference genes depending on CV variation. Accession numbers and the expression values from the transcriptome analysis for all 17 candidates are listed in S1 Table.

### RT-qPCR amplification efficiency and primer specificity

The RT-qPCR primers were designed using PrimerQuest for the 17 candidate reference genes to generate an amplicon ranging in size from 101 to 152 bp for each reference gene (Table 2). The PCR products were amplified using cDNA made from total RNA of *S*. *uncinata* gametophytes grown at 15°C as a template, and the specificity and accuracy of the primers were verified by 2% agarose gel electrophoresis (S2A Fig). The dissociation curves of each PCR product showed a unique melting peak of fluorescence signatures, suggesting that only gene-specific DNA fragments were amplified during the PCR reaction with primer pairs of each candidate gene (S2B Fig). The PCR efficiency of each primer pair was greater than 90%, and ranged from 94.17% (*TUB250*) to 104.26% (*GAPDH*); correlation coefficients (*R*^2^) of the standard curve were between 0.96 (*TUB250*) and 0.999 (*UK552*) (Table 2 and S3 Fig).

**Table 2:** Primer sequences and amplicon characteristics of 17 candidate reference genes for RT-qPCR analysis.

Gene group	Gene symbol	Accession number	Gene name	Primer sequence (Forward /Reverse)	Amplicon size (bp)	PCR efficiency (%)*	*R*^2^*
1	*60S897*	MG020635	60s ribosomal protein L23A	CTAGAGGCTTGGCCTGTTT	106	105.72	0.996
				TGACAAGGTAGGAAGCAATGA			
1	*E2*.*299*	MG020639	Ubiquitin-conjugating enzyme E2	CCAGTGATCTCACTCACATGC	124	101.05	0.998
				TGTCTATGGCAGTCCCTTCT			
1	*E3*.*528*	MG020640	E3 ubiquitin ligase	GCAAGTTGGCTGCTTTCATTT	109	102.17	0.993
				ACTTTAAGCGCTCACCAGAAG			
1	*EF1*.*278*	MG020641	Elongation factor 1-delta	CGCTTCTTCTTTCTGGGATACA	121	97.2	0.984
				GTGCAAGGTTGAACACACATAC			
1	*HIS*	MG020644	Histone H3.3 isoform X1	TGTCAACATATCCGAAGCTAGT	116	99.83	0.996
				TGAATAGAGTTCTCTTGTAGATTGC			
1	*tIF*	MG020647	Translation initiation factor eIF-2B	GGGTTCCTCGGTTCATGTT	136	99.79	0.985
				CAAGGTGGTAGCCCTGTATT			
2	*AKB*	MG020637	Adenylate kinase 4	GCAAGCAAGCCATCCTATTTG	101	100.25	0.994
				TGTTCAACAAGGCACACTTAAAC			
2	*HMT*	MG020643	Histone H3 methyltransferase complex	GAGGGTGGATCCAACAGATATT	135	101.39	0.995
				CCTGATTTCAGGACTCCTATCG			
2	*POB1*	MG020645	BTB/POZ domain-containing protein	AGCGTAGATTAGGTTTCCAGTTC	111	94.96	0.997
				TACGAAGCTGCGCAAAGT			
2	*SPT*	MG020646	Serine palmitoyltransferase 2	GAGACGCCCTACAGGATTTG	108	103.05	0.997
				ATTCCTATGCCTTTGCTCACT			
2	*UFD2*	MG020649	Ubiquitin-protein ligase	GAGTTGATATGCTGCAATGCC	103	95.82	0.997
				CTGGAAGCAACTCCTCACAA			
2	*UK552*	MG020650	Uncharacterized protein	AGATCTGACGTAAGCGTTGG	101	97.63	0.999
				GCACAGGTTCTGAGGATCAA			
3	*ACT5*	MG020636	Actin 5	AGCATGAAGATCAAGGTGGTG	101	96.03	0.982
				ATCCACATCTGCTGGAAAGTG			
3	*ARP9*	MG020638	Actin-related protein 9	GGCAGTGGCAGGAAGTATAG	117	99.54	0.996
				TCTGTTCGTCGTCCACAAAT			
3	*GAPDH*	MG020642	Glyceraldehyde-3-phosphate dehydrogenase	ATCTGTTGGGACCAGTTTCAG	109	104.26	0.998
				ACACGTGAGATCCAATGACAG			
3	*SuTub*	MG020651	Alpha tubulin	GCCACCATCAAGACCAAGA	152	95.34	0.992
				CTGGTGCTGTTCGAGATCAT			
3	*TUB250*	MG020648	Gamma tubulin	AGGTCAGTCCGTCCAGAA	105	94.17	0.96
				CCTGATGCCAGAGTCGTTAAA			

* Mean of 4 technical replicates

### Expression of the candidate reference genes

We prepared three experimental sets to verify the stability of candidate reference genes under various abiotic stress conditions. Since the *S*. *uncinata* culture samples used in this study were originally grown in the Antarctic, we selected cold and drought as the representative abiotic stressors for this species. To verify gene expression patterns under cold stress, axenic cultured *S*. *uncinata* gametophytes were transferred from 15°C, which is close to the optimum temperature for net photosynthesis [34], to 2°C, which is close to the average temperature of the summer season in the Barton Peninsula. After incubating for 1, 3, and 8 h, total RNAs were prepared from each condition. To verify gene expression patterns under drought stress, cultured *S*. *uncinata* gametophytes were transferred from agar plates to clean dishes followed by air-drying at room temperature for 6, 12, and 24 h. In addition to the cultured gametophytes, Antarctic field samples were prepared to test the applicability of the selected reference genes to naturally growing plants. For this reason, *S*. *uncinata* gametophyte plants were collected from three Antarctic field populations with a series of water contents, designated as W68, W48, and W32 and representing *S*. *uncinata* gametophytes with 68%, 48%, and 32% water contents, respectively; this comprised the WGF experimental setup (Table 1).

**Fig 1:**
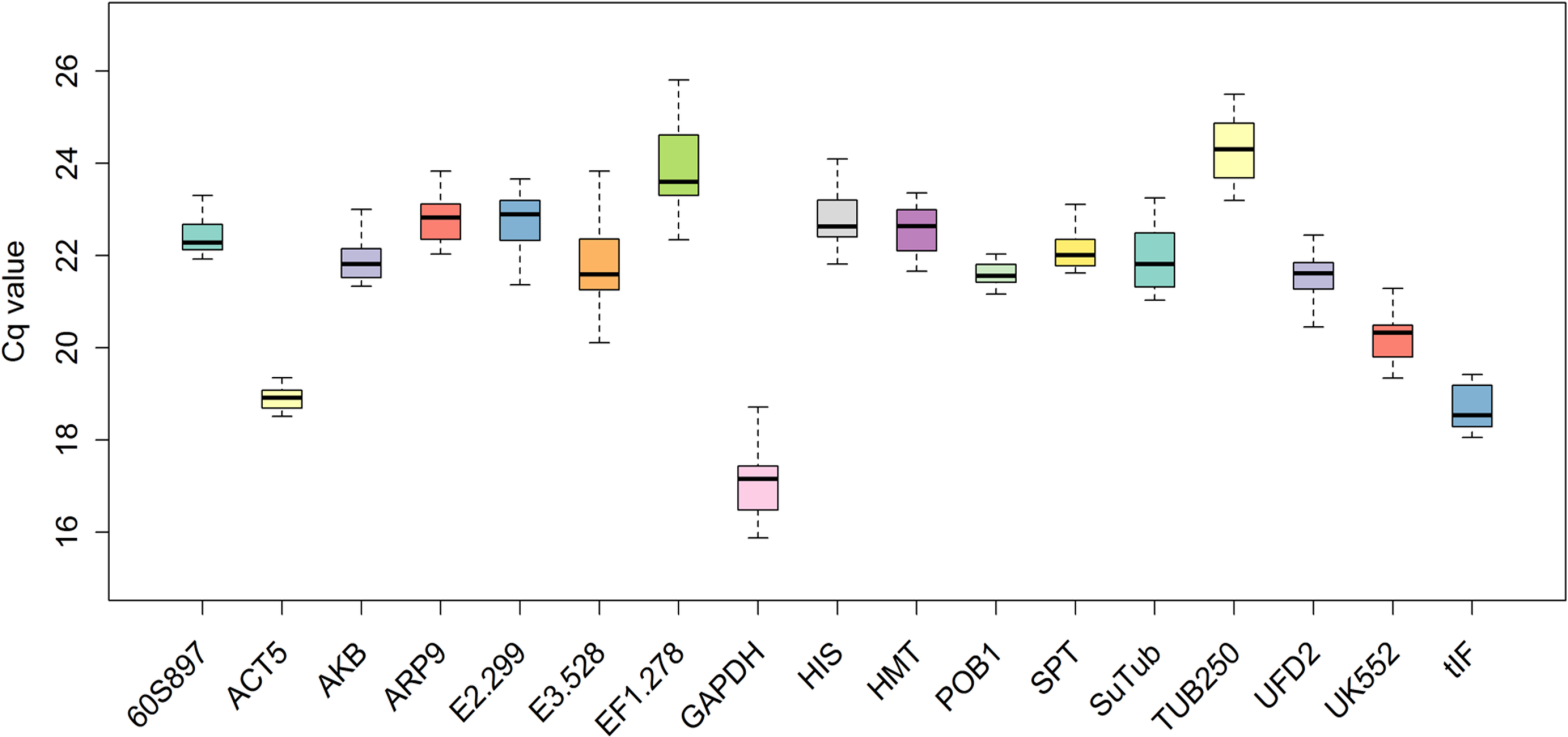
Distribution of the quantification cycle (Cq) values of the 17 candidate reference genes in *Sanionia uncinata* samples. The median Cq values for 11 samples are displayed in a boxplot indicating the interquartile range. Error bars represent the minimum and maximum Cq values and the middle panes show the mean values. Three biological replicates per sample and four technical replicates per biological replicate were used.

Using the 11 different RNA samples from the three experimental conditions, the expression levels of the 17 candidate reference genes were analyzed. Following amplification of each candidate gene with the 11 different RNA samples, the median Cq values of the 11 PCR amplifications, and the difference between the minimal and maximal Cq values, were obtained (Fig 1). The median Cq values for the reference genes ranged from 16 to 25. *Tub250* (24.25 ± 0.79) had the maximum median Cq value and *GAPDH* (17.18 ± 1.12) had the minimum, which indicates the lowest and highest expression, respectively. *ACT5* (18.95 ± 1.31) and *POB1* (21.5 ± 1.61) showed low gene expression variation, while *E3*.*528* (21.8 ± 3.98) and *SuTub* (21.8 ± 3.89) displayed high variation.

### Reference gene stability analysis with geNorm

**Fig 2:**
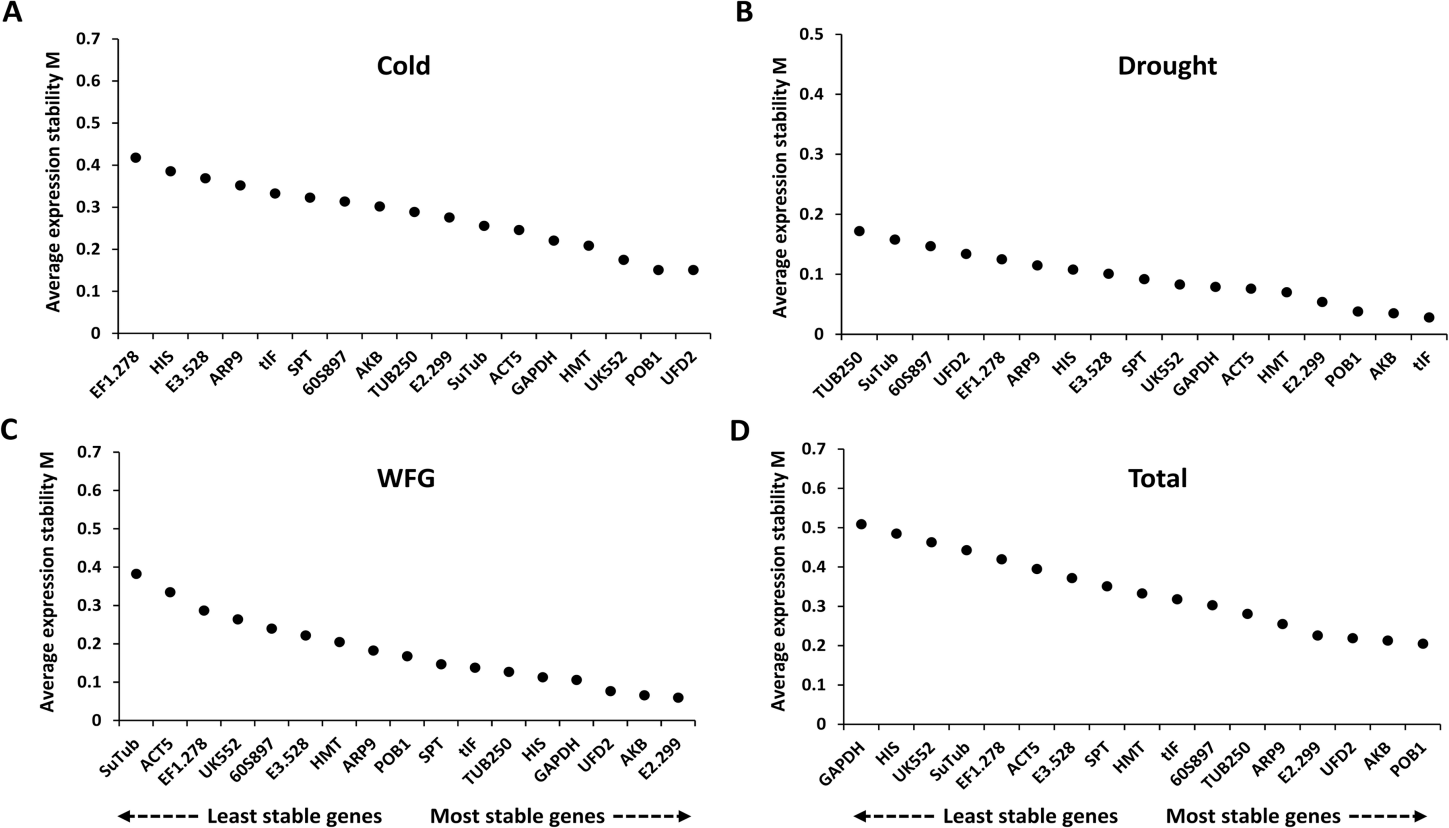
Expression stability values (M) of the candidate reference genes based on the geNorm algorithm. The cutoff for reference gene stability was proposed to be an M value of 0.5. Candidate reference genes were ranked in ascending order of expression stability, from the least stable on the left to the most stable gene on the right. (A) Cold experimental set, (B) drought experimental set, (C) WGF experimental set, and (D) total experimental set.

The gene expression stability of the 17 candidate reference genes was further evaluated by RT-qPCR Cq values and ranked using geNorm, NormFinder, and BestKeeper. The geNorm statistical algorithm determines gene expression stability (M) based on the average pairwise variations of all tested genes [12]. In this analysis, lower M values indicate more stable gene expression. When the geNorm algorithm was applied to the 17 candidate references from *S*. *uncinata*, they all exhibited M values lower than the suggested threshold value of 0.5 (Fig 2). Under the cold conditions, *POB1* and *UFD2* (M = 0.151 for both) were identified as the most stably expressed genes and *EF1*.*278* (M = 0.418) showed the least stability. Under drought conditions, *tIF* and *AKB* (M = 0.028 and 0.035, respectively) were among the most stable genes, while *TUB250* (M = 0.172) was the least stable. *E2*.*299* and *AKB* (M = 0.06 for both) were ranked as the most stable in the WGF experimental set, whereas *SuTub* (M = 0.383) was determined to be the least stable. When the 11 RNA samples from all three experimental sets were considered together, the most stable genes were *POB1* and *AKB* (M = 0.205 and 0.213, respectively), whereas the most unstable gene was *GAPDH* (M = 0.509) (Fig 2).

Notably, some candidate genes showed highly variable expression depending on the experimental conditions. For example, *GAPDH* had a low M value in the cold (M = 0.221), drought (M = 0.079), and WGF (M = 0.106) experimental sets (Fig 2A–2C), but showed the highest score when tested with the total set (M = 0.509) (Fig 2D). *HIS* had a low score under drought (M = 0.108) and WGF (M = 0.113) conditions, but exhibited a high score in the total set (M = 0.485) (Fig 2D). These results indicated that there could be a large difference in stability rankings between a single stressor and multiple stressors, underscoring the importance of validation of the reference genes in the intended experimental conditions.

**Fig 3:**
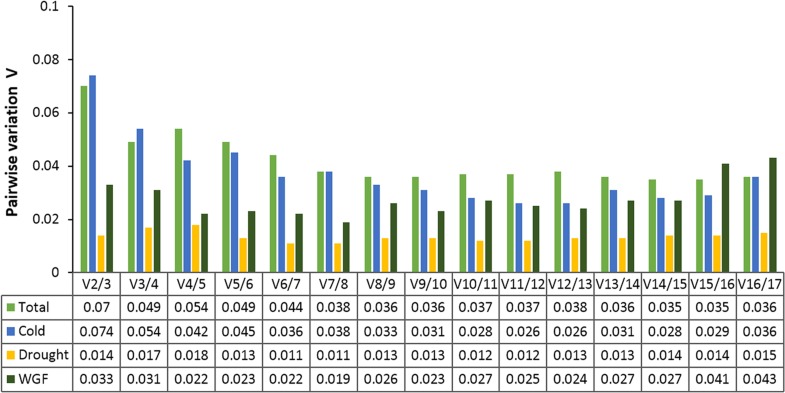
Pairwise variation (Vn/Vn+1) analysis of the candidate reference genes. The pairwise variation (Vn/Vn+1) was analyzed based on the geNorm algorithm to determine the optimal number of reference genes for accurate normalization. A cutoff value of 0.15 was set for the pairwise variation, as recommended by Vandesompele et al (2002) [12].

The geNorm also calculates a normalization factor to determine the optimal number of reference genes for accurate normalization, based on pairwise variations (Vn/n + 1) between each combination of sequential normalization factors. A cutoff value of 0.15 is the recommended threshold, which indicates that an additional reference gene will make no remarkable contribution to the normalization [12]. The V2/3 values in the total, cold, drought, and WGF experimental sets were less than 0.15 (Fig 3), suggesting that the two most stable reference genes are sufficient for accurate normalization.

### Reference gene stability analysis with NormFinder

**Fig 4:**
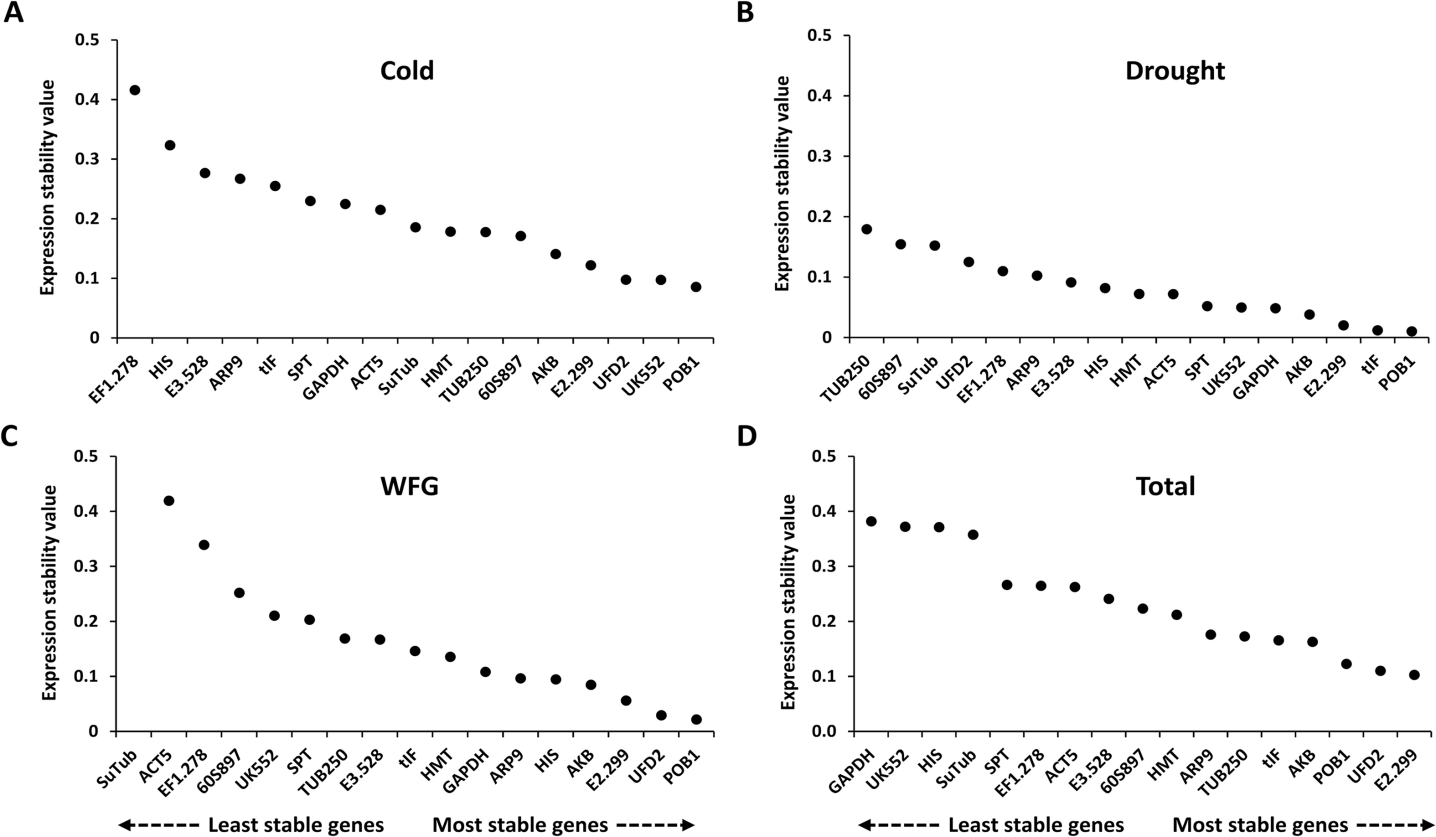
Expression stability values and ranking of the candidate reference genes based on the NormFinder algorithm. Candidate reference genes were ranked in ascending order of expression stability, from the least stable on the left to the most stable gene on the right. (A) Cold experimental set, (B) drought experimental set, (C) WGF experimental set, and (D) total experimental set.

The 17 candidate reference genes were further evaluated with the NormFinder algorithm to identify the optimal normalization genes under the different experimental conditions. NormFinder evaluates the stability of reference genes with the expression variations of candidate genes, using a model-based approach to rank candidate reference genes based on intra- and inter-group expression variation [28]. Similar to the geNorm analysis, lower stability values indicate more stable gene expression. While NormFinder predicted *POB1* as the most stable gene under the cold, drought, and total experimental set, *POB1* was the fourth most stable gene in the WGF experimental sets (Fig 4). Although the rankings of *POB1*, *UFD2*, and *AKB* varied, they were commonly predicted as the top five stable genes in all four experimental sets.

### Reference gene stability analysis with BestKeeper

BestKeeper uses the SD and CV of Cq values to calculate the expression stability of candidate reference genes [29]. When the expression stability of the 17 candidate genes was analyzed via BestKeeper (Table 3), two genes showing the lowest CV±SD values [*HMT* (1.69±0.38) and *AKB* (2.05±0.45)] were confirmed as the most stable genes in total experimental set. Under cold condition, *TUB250* and *AKB*, which showed the lowest CV±SD values of 2.07±0.51 and 2.49±0.54, respectively, were ranked as the top two most stable genes. The results under drought conditions revealed that *POB1* (CV±SD = 0.71±0.15) and *ARP9* (CV±SD = 0.86±0.19) were the most stable genes. Furthermore, in the WGF experimental set, *UFD2* (CV±SD = 0.17±0.04) and *SPT* (CV±SD = 0.20±0.05) were identified as the most stable genes by BestKeeper.

**Table 3:** Stability analysis of 17 candidate reference genes using BestKeeper.

Rank	Cold			Drought			WGF			Total		
	Gene	SD	CV	Gene	SD	CV	Gene	SD	CV	Gene	SD	CV
1	*TUB250*	0.51	2.07	*POB1*	0.15	0.71	*UFD2*	0.04	0.17	*HMT*	0.38	1.69
2	*AKB*	0.54	2.49	*ARP9*	0.19	0.86	*SPT*	0.05	0.20	*AKB*	0.45	2.05
3	*SuTub*	0.57	2.57	*AKB*	0.22	1.00	*GAPDH*	0.05	0.25	*POB1*	0.51	2.32
4	*ARP9*	0.60	2.68	*SuTub*	0.23	1.08	*60S897*	0.14	0.61	*ARP9*	0.57	2.50
5	*UFD2*	0.62	2.87	*GAPDH*	0.24	1.46	*HMT*	0.14	0.62	*E3*.*528*	0.57	2.64
6	*SPT*	0.68	3.07	*ACT5*	0.26	1.45	*POB1*	0.16	0.74	*E2*.*299*	0.58	2.53
7	*POB1*	0.70	3.22	*E2*.*299*	0.27	1.21	*E3*.*528*	0.18	0.82	*UFD2*	0.58	2.70
8	*E3*.*528*	0.70	3.26	*60S897*	0.30	1.39	*EF1*.*278*	0.19	0.75	*UK552*	0.58	2.89
9	*EF1*.*278*	0.71	3.00	*HMT*	0.30	1.33	*AKB*	0.22	1.00	*ACT5*	0.59	3.14
10	*HMT*	0.71	3.16	*UFD2*	0.30	1.44	*TUB250*	0.23	0.93	*TUB250*	0.60	2.45
11	*GAPDH*	0.71	4.27	*EF1*.*278*	0.31	1.36	*HIS*	0.23	0.97	*SPT*	0.63	2.88
12	*E2*.*299*	0.73	3.23	*UK552*	0.33	1.64	*ARP9*	0.24	1.04	*SuTub*	0.66	2.98
13	*60S897*	0.73	3.27	*TUB250*	0.34	1.46	*SuTub*	0.24	1.05	*60S897*	0.72	3.21
14	*ACT5*	0.73	3.80	*HIS*	0.35	1.57	*E2*.*299*	0.27	1.15	*GAPDH*	0.72	4.20
15	*UK552*	0.80	4.10	*SPT*	0.38	1.78	*UK552*	0.34	1.65	*EF1*.*278*	0.80	3.38
16	*HIS*	0.83	3.64	*E3*.*528*	0.70	3.29	*tIF*	0.35	1.92	*HIS*	0.90	3.94
17	*tIF*	1.15	6.18	*tIF*	0.91	5.34	*ACT5*	0.43	2.22	*tIF*	0.94	5.26

### Comprehensive reference gene selection

To determine the most stable genes predicted by the three different algorithms, we integrated the results from geNorm, NormFinder, and BestKeeper to determine comprehensive ranking for each candidate reference gene based on the geometric mean of rankings from three algorithms [35]. As shown in Table 4, *POB1* and *UFD2* were recommended as the most stable reference genes under cold conditions. *AKB* and *POB1* were chosen as the most stable genes for the drought and all experimental sets. Moreover, *UFD2* and *AKB* were predicted as the most stable genes in the WGF experimental set. In reverse, we could select the least stable genes for each experimental set. Overall, traditional housekeeping genes belonging to the gene group 3, such as *ACT5*, *GAPDH*, *SuTub*, and *TUB250*, showed low stability in experimental sets tested (Table 4). Interestingly, *UFD2* was ranked to the most stable genes in the cold and WGF experimental sets, however, it was one of the two least stable genes in the drought experimental set.

**Table 4:** Comprehensive stability ranking of the candidate reference genes.

**Method**	**1**	**2**	**3**	**4**	**5**	**6**	**7**	**8**	**9**
**Ranking order for the cold experimental set (Better—Good—Average)**									
geNorm	*POB1/UFD2*		*UK552*	*HMT*	*GAPDH*	*ACT5*	*SuTub*	*E2*.*299*	*TUB250*
NormFinder	*POB1*	*UK552*	*UFD2*	*E2*.*299*	*AKB*	*60S897*	*TUB250*	*HMT*	*SuTub*
BestKeeper	*TUB250*	*AKB*	*SuTub*	*ARP9*	*UFD2*	*SPT*	*POB1*	*E3*.*528*	*EF1*.*278*
Comprehensive ranking	*POB1*	*UFD2*	*TUB250*	*UK552*	*AKB*	*SuTub*	*HMT*	*E2*.*299*	*GAPDH*
**Ranking order for the drought experimental set (Better—Good—Average)**									
geNorm	*tIF*	*AKB*	*POB1*	*E2*.*299*	*HMT*	*ACT5*	*GAPDH*	*UK552*	*SPT*
NormFinder	*POB1*	*tIF*	*E2*.*299*	*AKB*	*GAPDH*	*UK552*	*SPT*	*ACT5*	*HMT*
BestKeeper	*POB1*	*ARP9*	*AKB*	*SuTub*	*GAPDH*	*ACT5*	*E2*.*299*	*60S897*	*HMT*
Comprehensive ranking	*POB1*	*AKB*	*tIF*	*E2*.*299*	*GAPDH*	*ARP9*	*ACT5*	*HMT*	*UK552*
**Ranking order for the WGF experimental set (Better—Good—Average)**									
geNorm	*E2*.*299*	*AKB*	*UFD2*	*GAPDH*	*HIS*	*TUB250*	*tIF*	*SPT*	*POB1*
NormFinder	*POB1*	*UFD2*	*E2*.*299*	*AKB*	*HIS*	*ARP9*	*GAPDH*	*HMT*	*tIF*
BestKeeper	*UFD2*	*SPT*	*GAPDH*	*60S897*	*HMT*	*POB1*	*E3*.*528*	*EF1*.*278*	*AKB*
Comprehensive ranking	*UFD2*	*AKB*	*E2*.*299*	*POB1*	*GAPDH*	*SPT*	*HIS*	*HMT*	*TUB250*
**Ranking order for the total experimental set (Better—Good—Average)**									
geNorm	*POB1*	*AKB*	*UFD2*	*E2*.*299*	*ARP9*	*TUB250*	*60S897*	*tIF*	*HMT*
NormFinder	*E2*.*299*	*UFD2*	*POB1*	*AKB*	*tIF*	*TUB250*	*ARP9*	*HMT*	*60S897*
BestKeeper	*HMT*	*AKB*	*POB1*	*ARP9*	*E3*.*528*	*E2*.*299*	*UFD2*	*UK552*	*ACT5*
Comprehensive ranking	*POB1*	*AKB*	*E2*.*299*	*UFD2*	*HMT*	*ARP9*	*TUB250*	*E3*.*528*	*tIF*
**Method**		**10**	**11**	**12**	**13**	**14**	**15**	**16**	**17**
**Ranking order for the cold experimental set (Better—Good—Average)**									
geNorm		*AKB*	*60S897*	*SPT*	*tIF*	*ARP9*	*E3*.*528*	*HIS*	*EF1*.*278*
NormFinder		*ACT5*	*GAPDH*	*SPT*	*tIF*	*ARP9*	*E3*.*528*	*HIS*	*EF1*.*278*
BestKeeper		*HMT*	*GAPDH*	*E2*.*299*	*60S897*	*ACT5*	*UK552*	*HIS*	*tIF*
Comprehensive ranking		*ARP9*	*ACT5*	*60S897*	*SPT*	*E3*.*528*	*EF1*.*278*	*tIF*	*HIS*
**Ranking order for the drought experimental set (Better—Good—Average)**									
geNorm		*E3*.*528*	*HIS*	*ARP9*	*EF1*.*278*	*UFD2*	*60S897*	*SuTub*	*TUB250*
NormFinder		*HIS*	*E3*.*528*	*ARP9*	*EF1*.*278*	*UFD2*	*SuTub*	*60S897*	*TUB250*
BestKeeper		*UFD2*	*EF1*.*278*	*UK552*	*TUB250*	*HIS*	*SPT*	*E3*.*528*	*tIF*
Comprehensive ranking		*SPT*	*SuTub*	*HIS*	*E3*.*528*	*EF1*.*278*	*60S897*	*UFD2*	*TUB250*
**Ranking order for the WGF experimental set (Better—Good—Average)**									
geNorm		*ARP9*	*HMT*	*E3*.*528*	*60S897*	*UK552*	*EF1*.*278*	*ACT5*	*SuTub*
NormFinder		*E3*.*528*	*TUB250*	*SPT*	*UK552*	*60S897*	*EF1*.*278*	*ACT5*	*SuTub*
BestKeeper		*TUB250*	*HIS*	*ARP9*	*SuTub*	*E2*.*299*	*UK552*	*tIF*	*ACT5*
Comprehensive ranking		*ARP9*	*60S897*	*E3*.*528*	*tIF*	*EF1*.*278*	*UK552*	*SuTub*	*ACT5*
**Ranking order for the total experimental set (Better—Good—Average)**									
geNorm		*SPT*	*E3*.*528*	*ACT5*	*EF1*.*278*	*SuTub*	*UK552*	*HIS*	*GAPDH*
NormFinder		*E3*.*528*	*ACT5*	*EF1*.*278*	*SPT*	*SuTub*	*HIS*	*UK552*	*GAPDH*
BestKeeper		*TUB250*	*SPT*	*SuTub*	*60S897*	*GAPDH*	*EF1*.*278*	*HIS*	*tIF*
Comprehensive ranking		*60S897*	*ACT5*	*SPT*	*UK552*	*EF1*.*278*	*SuTub*	*HIS*	*GAPDH*

**Fig 5:**
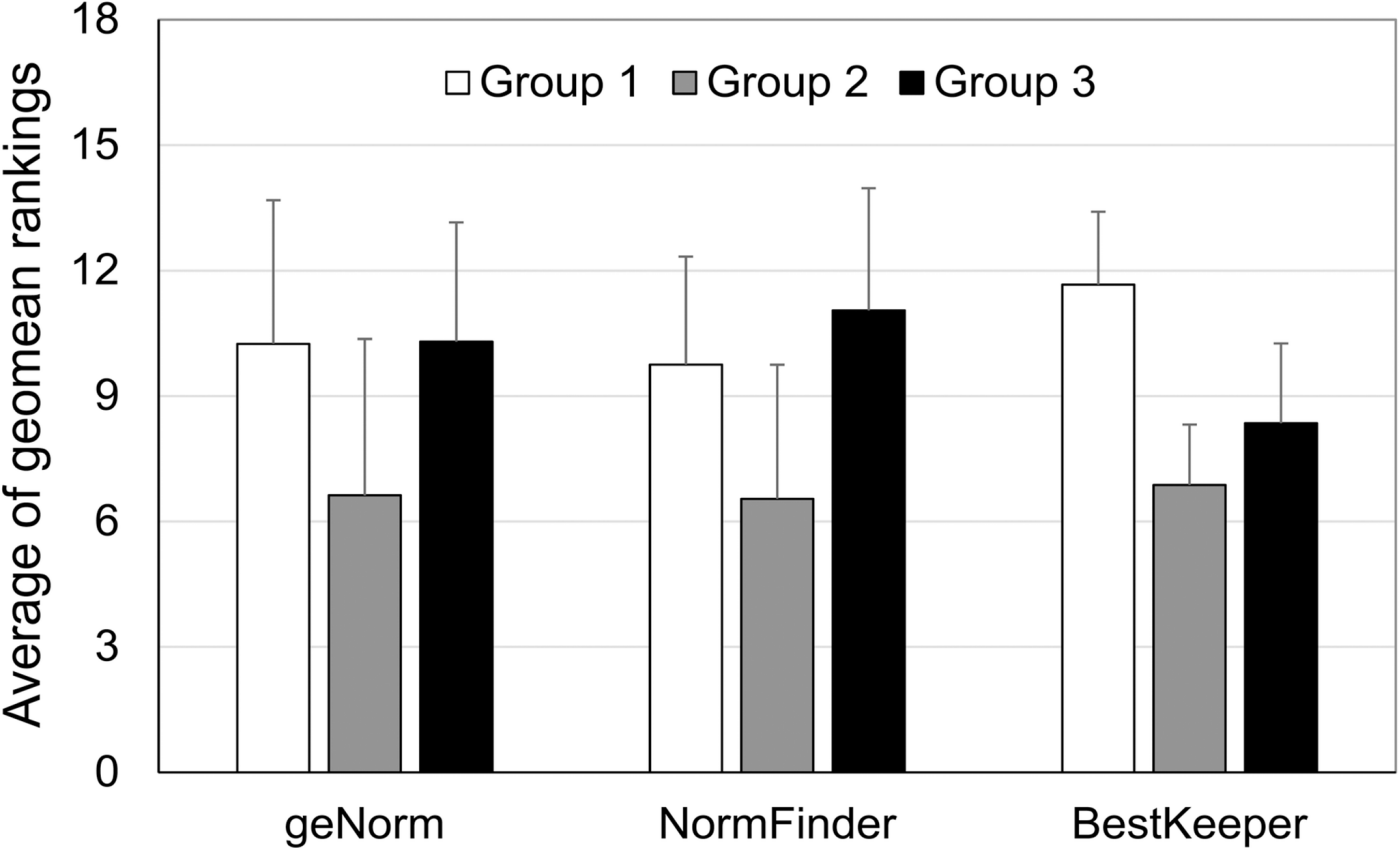
Distribution of average geomean rankings for candidate reference genes depending of the gene group. Values were obtained by calculating an average comprehensive ranking for the genes in the gene group 1, 2, and 3, respectively. Four datasets from different experimental sets were used, and error bars represent standard deviation.

To better clarify the pattern of comprehensive rankings of candidates depending on the gene group, we calculated averages of geomean values from three different algorithms for each gene group. As shown in Fig 5, although the slight difference between the values of the gene group 1 and the gene group 3, the gene group 2 showed constantly lowest values by all three algorithms, irrespective of the program used. This result indicates that genes in the group 2, which were novel genes with the low CV of transcriptome, were ranked higher and were more stable compared to the other housekeeping genes with low CV (group 1) or high CV (group 3).

### Validation of the recommended reference genes

**Fig 6:**
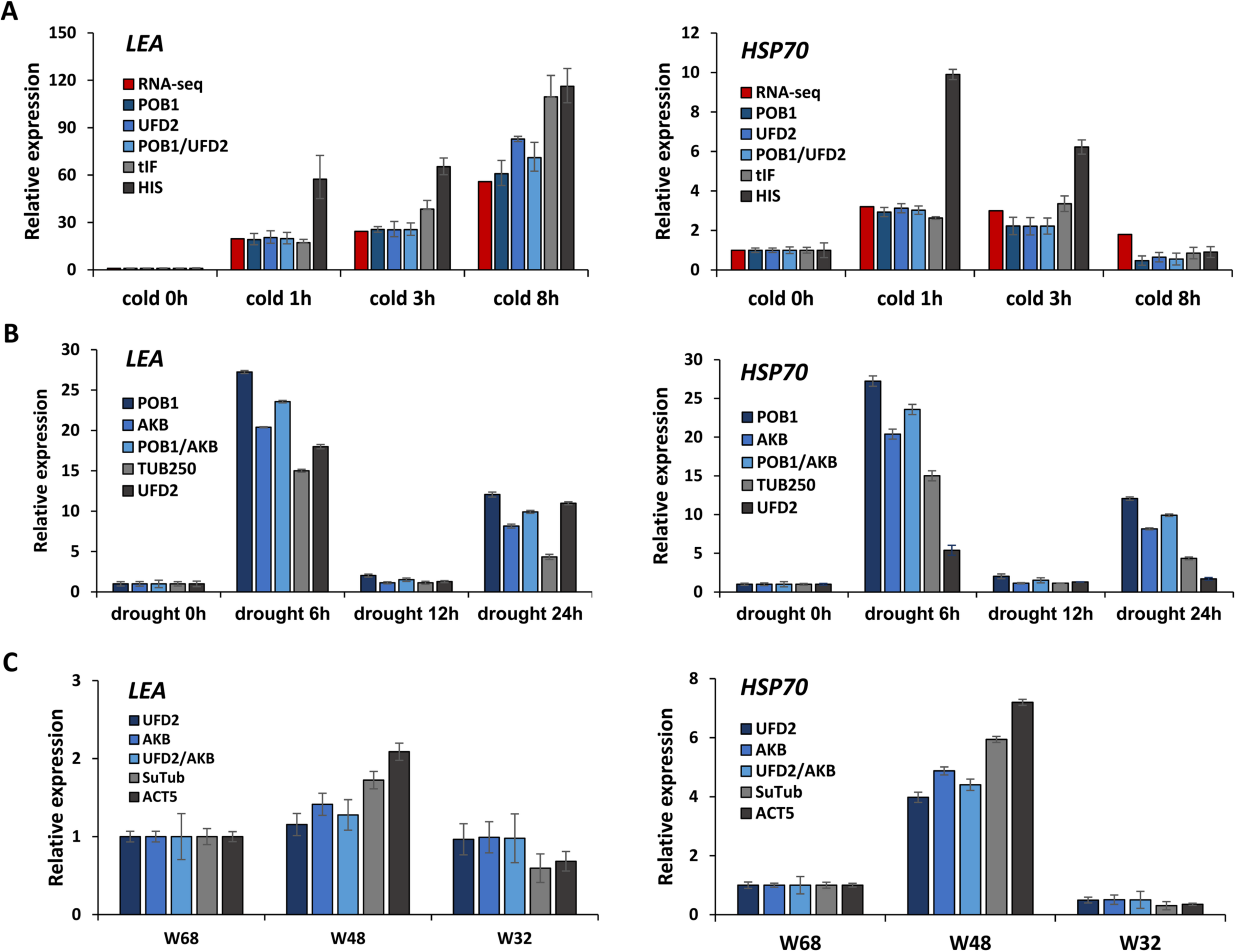
Relative expression levels of the late embryogenesis abundant (*LEA*) and heat shock protein 70 (*HSP70*) genes under different stress conditions when the selected reference genes were used for normalization. (A) *LEA* and *HSP70* expression in the cold experimental set of *S*. *uncinata* was normalized using a combination of the two most stable genes *POB1* and *UFD2*, and the least stable genes *HIS* and *tIF*. Red bar indicates fold-change values of gene expression, which were calculated based on the normalized FPKM from the RNA-Seq analysis of the cold experimental set (S2 Table). (B) The expression of *LEA* and *HSP70* in the drought experimental set of *S*. *uncinata* was normalized using a combination of the two most stable genes *POB1* and *AKB*, and the least stable genes *UFD2* and *TUB250*. (C) The expression of *LEA* and *HSP70* in the WGF experimental set of *S*. *uncinata* was normalized using a combination of the two most stable genes *UFD2* and *AKB*, and the least stable genes *SuTub* and *ACT5*. Error bars represent the standard deviation of the mean of three biological replicates.

To validate the best-ranked candidate reference genes, the selected genes were used to normalize the expression patterns of the genes *LEA* and *HSP70*. Since *LEA* and *HSP70* are known to be upregulated by various environmental [35–37], they are good markers of abiotic stress responses in plants. We identified a homologous sequence for each gene in the *S*. *uncinata* transcriptome dataset of this study. The *LEA* gene identified from *S*. *uncinata* showed 47% sequence identity with *Triticum aestivum LEA3* (AAN74639) at both the nucleotide and amino acid levels. In addition, the transcript levels of the *S*. *uncinata LEA* homolog exhibited large, positive fold changes under cold conditions: an approximately 20-, 25-, and 60-fold increase after 1, 3, and 8 h, respectively. The *HSP70* gene from *S*. *uncinata* showed high sequence identity at both the nucleotide and amino acid levels (81.6% and 92%, respectively) with the *Bryum argenteum HSP70* gene (KP087877). The transcript levels of the *S*. *uncinata HSP70* homolog exhibited positive fold changes under cold conditions: a 4.8-, 5.6-, and 1.9-fold increase after 1, 3, and 8 h, respectively. Thus, we concluded that the *LEA* and *HSP70* homologs from *S*. *uncinata* are *bona fide* stress-responsive genes suitable for reference gene evaluations (Fig 6A).

For the quantification of target gene expression by RT-qPCR in the cold, drought, and WGF experimental sets, we used the top two most stable genes and the two least stable genes as references. Induction patterns similar to RNA-Seq under cold stress condition were observed when the Cq values of marker genes were normalized to the best candidate genes *POB1* or *UFD2* or both of *POB1/UFD2*, with only small variations in fold change values in each normalization (Fig 6A). On the other hand, when data normalization was performed with the least stable genes, the values differed substantially. For example, the ranges of fold change for *LEA* was 20–60 and 60–120 when *POB1* and *HIS* were used as the reference gene, respectively. In the drought and WGF experimental sets, the normalized expression levels of the target genes also differed from the results obtained with the most stable genes (*POB1* and *AKB* for drought; *UFD2* and *AKB* for WGF) and the most variable genes (*UFD2* and *TUB250* for drought; *SuTub* and *ACT5* for WGF) (Fig 6B and 6C). Overall, our validation suggested that the expression profiles of target genes are strongly affected by the choice of reference gene.

## Discussion

The analysis of gene expression is a common strategy used to identify genes involved in a specific biological process. As data normalization is an essential step in gene expression analysis, the accuracy of RT-qPCR data can be greatly improved by the inclusion of a reference gene whose transcriptional levels are maintained across different experimental conditions [38]. Therefore, the selection of suitable reference genes for RT-qPCR is crucial for analyzing the expression of key genes correlated with various biological conditions. The present study assessed the stability of candidate reference genes for *S*. *uncinata* under different stress conditions.

We selected 17 candidate reference genes for *S*. *uncinata* under several experimental conditions, especially for representative abiotic stresses such as cold and drought, from axenic culture to Antarctic field samples. The expression stability of the candidate reference genes was evaluated using geNorm, NormFinder, and BestKeeper. The results of our conjoint analysis could provide a reliable basis for the selection of reference genes for RT-qPCR analysis with various treatments. A set of reference genes varied slightly in ranking, although some genes were consistently found in the upper ranks of the list of stably expressed genes (Table 4).

When we compared results from the three different analysis algorithms, the best reference genes differed depending on the experimental set (Table 4). *POB1* and *UFD2* were the most stable reference genes in the cold experimental set, while *POB1* and *AKB* were the most stable in the drought experimental set. In the WGF experimental set, *UFD2* and *AKB* showed maximum stability. Overall, the three novel reference genes, *AKB*, *POB1*, and *UFD2*, showed the most robust stability in multiple experimental sets, while traditionally used reference genes, such as *ACT5*, *GAPDH*, *SuTub*, and *TUB250*, were the least stable (Table 4). Also, when we compared the distribution of comprehensive rankings of the gene groups, the novel candidates genes in the group 2 were constantly more stable than the other two groups (Fig 5), indicating that traditionally used reference genes are often not the best choice for the normalization of RT-qPCR data in mosses under conditions of abiotic stress. The usefulness of transcriptome data for identifying robust normalizers is consistent with previous reports on the validation of novel reference genes based on transcriptome data [39, 40]. However, it is still necessary to validate the gene reliability individually even after the candidate gene selection from transcriptome data.

To validate the suitability of the identified reference genes, the expression patterns of the genes *LEA* and *HSP70* were investigated under different experimental conditions using different reference genes. The data once again showed that reference genes play an essential role in the normalization of RT-qPCR data, and the use of inappropriate reference genes may produce unreliable results. Significant differences were observed in the expression patterns of the target genes, *LEA* and *HSP70*, between the normalized values with the two most stable genes and the two least stable genes in most of the experimental conditions tested (Fig 6).

## Conclusion

In this study, we evaluated the expression stability of 17 candidate reference genes suitable for *S*. *uncinata* under a wide range of environmental conditions using three widely used applications, and the comprehensive stability ranking by the geometric mean. We found the best reference genes for the gene expression studies in abiotic stress responses of *S*. *uncinata*; *POB1* and *UFD2* for cold stress, *POB1* and *AKB* for drought stress, and *UFD2* and *AKB* for populations from different water gradients in the Antarctic field. For the preliminary study on the general abiotic stresses, *POB1* and *AKB* pair should be the first choice. In addition, we also confirmed that the novels genes with the low CV from the transcriptome data were more stable than the traditionally used reference genes. The stable reference genes for abiotic stresses identified in this study will facilitate future expression studies in mosses under various abiotic stresses.

## Supporting information

S1 Fig Agarose gel electrophoresis showing total RNA extracted from *S. uncinata* under various conditions. (A) cold stress, (B) drought stress, (C) water gradients in the field.
(PDF)

S2 Fig Verification of primer specificity for reverse transcription quantitative real-time polymerase chain reaction (RT-qPCR) amplification. (A) Agarose gel (2.0%) showing the amplified products of the 17 candidate reference genes at the expected sizes. (B) Melting curves of the 17 candidate reference genes.
(PDF)

S3 Fig Amplification efficiencies of primers used in this study. (PDF)

S1 File The contig sequences for candidate genes. The regions for RT-qPCR amplification were highlighted in red color.
(DOCX)

S1 Table Expression values of the 17 candidate genes of *S. uncinata* from the transcriptome under cold stress. (XLSX)

S2 Table Expression values of *LEA* and *Hsp70* in *S. uncinata* from the transcriptome under cold stress. (XLSX)
