# Supplementary figures and images for: Identification of reference genes for RT-qPCR in the Antarctic moss Sanionia uncinata under abiotic stress conditions
Source: PLoS One. 2018 Jun 19;13(6):e0199356. doi: 10.1371/journal.pone.0199356 (PMC6007896; doi:10.1371/journal.pone.0199356)

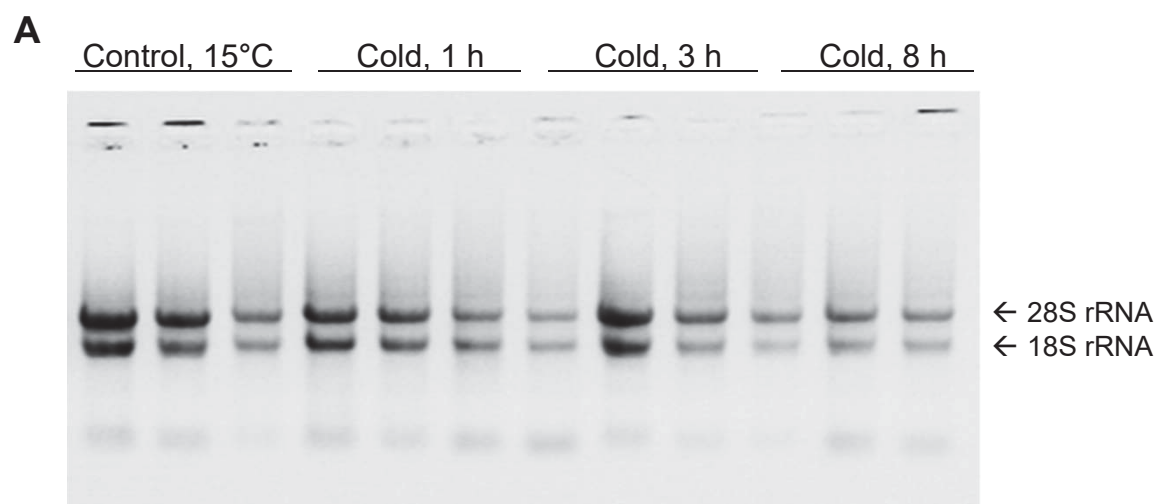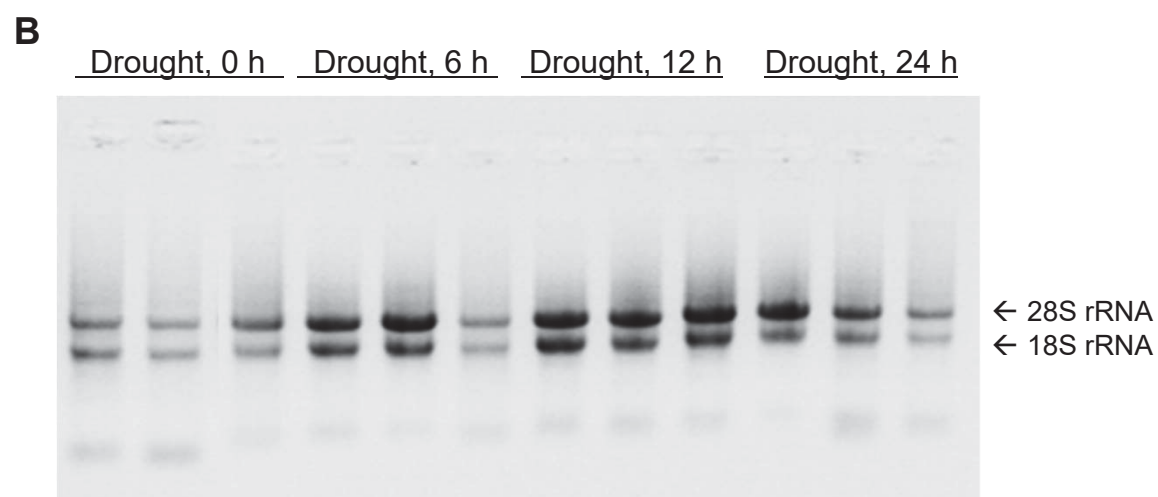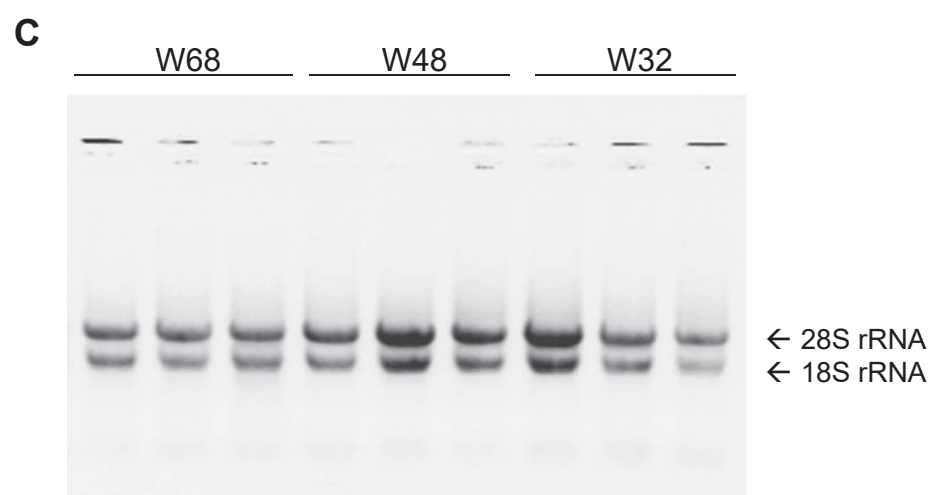

Supplement: S1 Fig — (A) cold stress, (B) drought stress, (C) water gradients in the field. (PDF) [file pone.0199356.s001.pdf]

**A**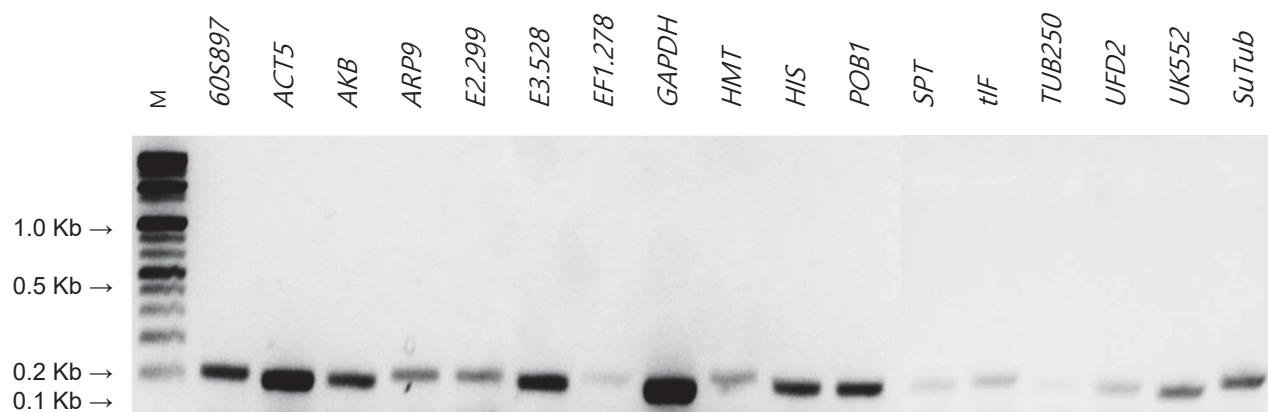**B**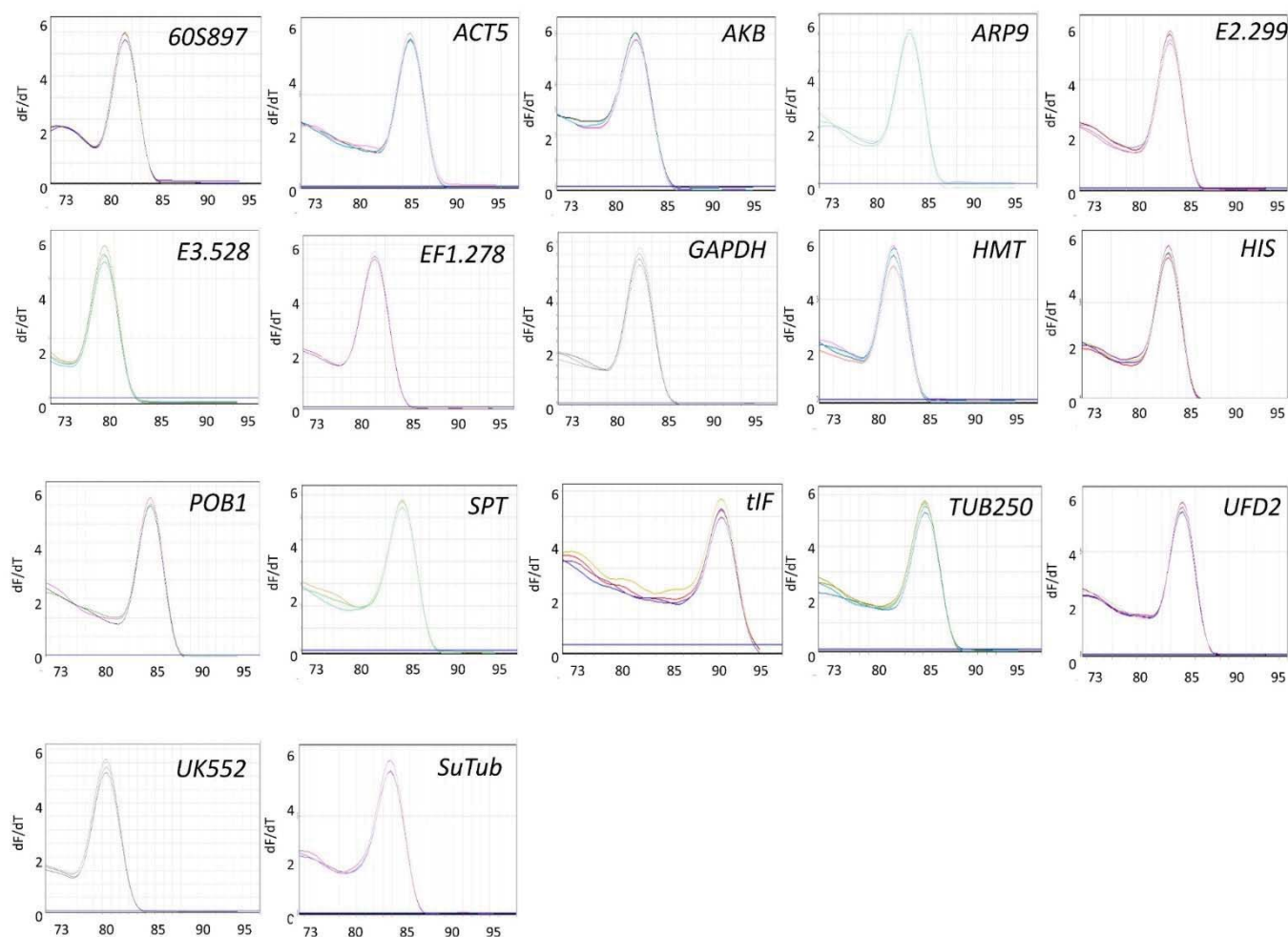

Supplement: S2 Fig — (A) Agarose gel (2.0%) showing the amplified products of the 17 candidate reference genes at the expected sizes. (B) Melting curves of the 17 candidate reference genes. (PDF) [file pone.0199356.s002.pdf]
