# Supplementary material for: Identification of reference genes for RT-qPCR in the Antarctic moss Sanionia uncinata under abiotic stress conditions
Source: PLoS One. 2018 Jun 19;13(6):e0199356. doi: 10.1371/journal.pone.0199356 (PMC6007896; doi:10.1371/journal.pone.0199356)
Supplement: S3 Fig — (PDF) [file pone.0199356.s003.pdf]

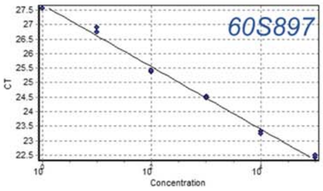

• standard
PCR efficiency(%)=105.72
R^2=0.996

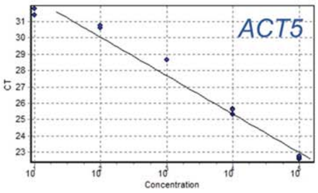

• standard
PCR efficiency(%)=96.03
R^2=0.982

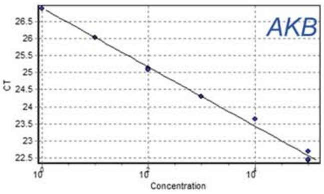

• standard
PCR efficiency(%)=100.25
R^2=0.994

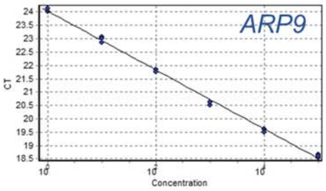

• standard
PCR efficiency(%)=99.54
R^2=0.996

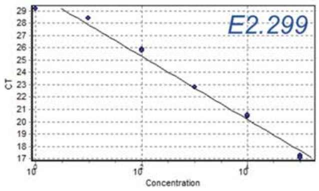

• standard
PCR efficiency(%)=101.05
R^2=0.998

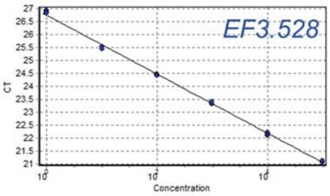

• standard
PCR efficiency(%)=102.17
R^2=0.993

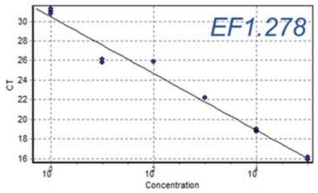

• standard
PCR efficiency(%)=97.2
R^2=0.984

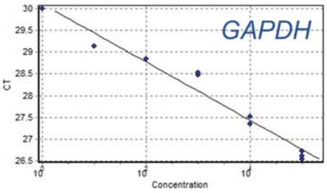

• standard
PCR efficiency(%)=104.26
R^2=0.998

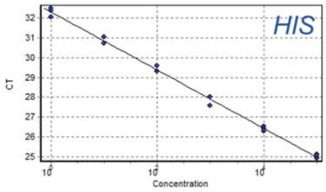

• standard
PCR efficiency(%)=99.83
R^2=0.996

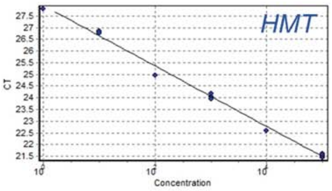

• standard
PCR efficiency(%)=101.39
R^2=0.995

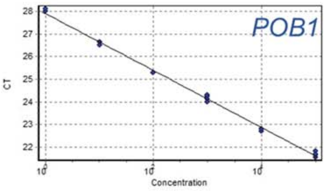

• standard
PCR efficiency(%)=94.96
R^2=0.997

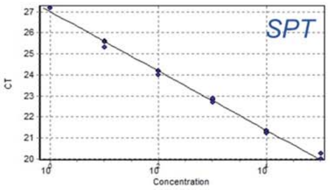

• standard
PCR efficiency(%)=103.05
R^2=0.997

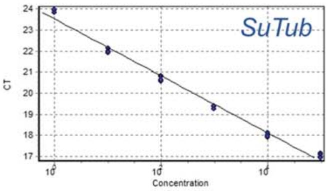

• standard
PCR efficiency(%)=95.34
R^2=0.992

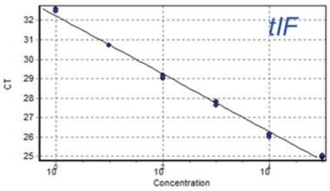

• standard
PCR efficiency(%)=99.79
R^2=0.985

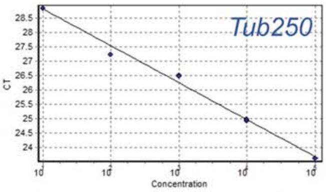

• standard
PCR efficiency(%)=94.14
R^2=0.96

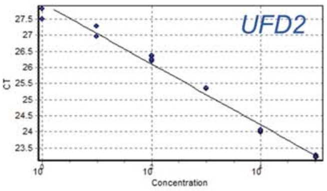

• standard
PCR efficiency(%)=95.82
R^2=0.997

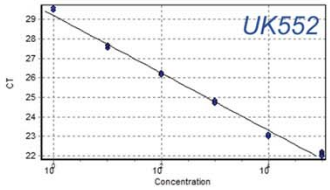

• standard
PCR efficiency(%)=97.63
R^2=0.999
