# Supplementary material for: Identification of reference genes for RT-qPCR in the Antarctic moss Sanionia uncinata under abiotic stress conditions
Source: PLoS One. 2018 Jun 19;13(6):e0199356. doi: 10.1371/journal.pone.0199356 (PMC6007896; doi:10.1371/journal.pone.0199356)
Supplement: S1 File — The regions for RT-qPCR amplification were highlighted in red color. (DOCX) [file pone.0199356.s004.docx]

Supporting Information

Identification of reference genes for quantitative real-time PCR in the Antarctic moss *Sanionia uncinata* under abiotic stress conditions

Mira Park^1,3^, Soon Gyu Hong^2,4^, Hyun Park^1,4^, Byeong-ha Lee^3*^, and Hyoungseok Lee^1,4*^

^1^Unit of Polar Genomics, Korea Polar Research Institute, Incheon, South Korea

^2^Division of Life Sciences, Korea Polar Research Institute, Incheon, South Korea

^3^Department of Life Sciences, Sogang University, Seoul, South Korea

^4^Polar Science, University of Science & Technology, Daejeon, South Korea

*Corresponding authors

Email: [soulaid@kopri.re.kr](mailto:soulaid@kopri.re.kr) (HL); [byeongha@sogang.ac.kr](mailto:byeongha@sogang.ac.kr) (B-hL)

## S1 File. The contig sequences for candidate genes. The regions for RT-qPCR amplification were highlighted in red color.

>60S897 MG020635

ATGGCTCCTCCACCTTCGAAGGTGGCCAAGCCGGCGTCGGCGTCGGCCAAGGCGACGAAGGCCGCGAAGGCTTTGAAGACTTCCACGAAGCCCGTGAAGAAGCTGTGGCGCAAGGTGCGAACGTCCGTGACGTTCCATCGCCCGAAGACGCTGAAGCGCGCGAGGGCGCCCAAGTACCCGCGCCTGAGTGCGCCCACCCGCAGCAAGCTCGACCACTACGAGGTGCTCAAGTACCCGCTGACCACGGARTCCGCCATGAAGAAGATCGAGGACAACAACACGCTGGTGTTCATCGTGGACGTGCGCGCGGACAAGAAGAAGATCAAGGATGCCGTGAAGAAGATGTACGACATTCAGACGAAGAAGGTCAACACGTTGATCAGGCCTGACGGAAGTAAGAAGGCATATGTTAGACTGACGGCAGATTACGATGCACTTGATGTGGCCAACAAGATTGGTATCATCTAGAATGGTGCTAATTATTCCATTTTGTGATCCTATCAATCTAGAGGCTTGGCCTGTTTTCTGTAGGGCTTTAGGCTCCTCTTCCTTAGACCATTTTTCGTCCATACTTCGAAAATTTGCTTTTATCATTGCTTCCTACCTTGTCAATTGTACGTTTTTATTCCTCTGTGACCCTAAGATGGGCTTTACAACCATGATCCATAATCCAGTAGTAGCACTTTATATTGTGGTTCTCGTTTTCGTTTAAAAATGGTCCTTCAGCATTTAGGCTTTCGGTATTTGCACCTTACTGAAGATGTATTTACAAACGGC

>ACT5 MG020636

ATGGCTGATGGTGAGGATGTTCAGCCTTTGGTGTGCGACAATGGATCCGGAATGGTCAAGGCCGGTTTCGCTGGAGATGATGCTCCTCGTGCTGTGTTTCCCAGTATTGTTGGTCGCCCAAGGCACACCGGTGTGATGGTGGGCATGGGACAGAAGGACGCGTACGTGGGCGACGAGGCTCAGTCGAAGAGGGGTATCCTGACTCTAAAGTATCCCATTGAGCACGGAGTGGTGACGAATTGGGACGACATGGAGAAGATCTGGCACCATACCTTCTACAACGAGTTGCGTGTGGCTCCGGAGGAGCACCCTGTTCTGCTGACCGAGGCTCCTCTGAACCCTAAGGCGAACAGGGAGAAGATGACCCAGATCATGTTCGAGACGTTCAATGTTCCCGCCATGTACGTGGCCATTCAGGCCGTGCTTTCGCTGTACGCCAGTGGCCGAACCACGGGTATTGTGTTGGACAGCGGAGATGGTGTGACCCACACGGTGCCCATCTACGAGGGCTACGCTTTGCCCCACGCCATCCTTCGTTTGGATTTGGCCGGTCGTGACTTGACKGACGCCCTGATGAAGATCCTGACGGARCGCGGTTACTCTTTCACGACGACTGCGGAGCGTGAAATCGTGCGCGACATGAAAGAGAAGCTTGCKTATGTCGCCATTGACTTCGAGCAGGAGCTGGACACGGCYCGCAGCAGYTCGTCGTTGGAGAAGAGCTACGAGTTGCCTGATGGCCAGGTGATCACCATCGGTGCTGAACGTTTCCGGTGYGCGGAGGTTYTGTTCAATCCGTCTCTGATCGGGATGGAGGCAGCGGGCATTCACGAGACTACYTACAATTCGATCATGAAGTGCGACGTGGATATCCGTAAGGATCTGTACGGCAACATCGTGTTGTCTGGCGGTTCGACGATGTTCCCTGGCATTGCGGACCGTATGAGCAAGGAGATCACCGCGTTAGCCCCAAGCAGCATGAAGATCAAGGTGGTGGCTCCTCCGGAGAGGAAGTACAGTGTGTGGATCGGAGGATCGATCTTGGCTTCACTGAGCACTTTCCAGCAGATGTGGATTGCCAAGAGTGAGTATGATGAGTCAGGCCCCTCGATTGTGCACAGAAAACGACAGGCGGACTCTTCTTTCCGAATCGATATTCCAGCAATTTTGGATACAAAGTTGGAAATACAGTATCCCCCCCTTGTTGACTATTTTATTTTATCACCTGCCTACTGCTCGGAGAAATTACCAGTGACGGGAGAAGATGGAGTGAAAACCAAAAACCACTAA

>AKB MG020637

CTTGTTCCCTTTCGCCCGTCCTCTRCCTCCCCCGATCGCCATGGCCGCCGCCCAGCTCGAGGATGTCCCCAACGACACCCTCTTCCACGAGGTCCTGCGCCGCATGAAGTGCGCCCCCAAGAGCGAGAAGCGCCTCATCCTCGTCGGTCCTCCGGGATGTGGGAAGGGAACGCAGTCGCCCATTCTCAAGGAAGAGCATTGCCTGTGTCACTTGGCCACGGGTGACATGTTGCGAGCGGCAGTCGCGCAGAAGACGCCGCTCGGATTAGAAGCTAAGGCGGCCATGGATAAGGGCGCATTGGTGTCCGATGACTTGGTGGTGGGCATCATTGACGAGGCGATGAAGAAGCCGTCGTGCACCAAGGGCTTTATCTTGGATGGTTTCCCACGCACTGTCGTGCAAGCTCAGAAGTTGGATGCTGCTCTTGGGAAGCAGGGTGTAAAGATCGACAAGGTGTTGAACTTTGAGATTGACGATTCCATTCTGGAGGAGCGGATTACAGGCCGGTGGATCCACCCTGACAGTGGCCGTTCTTACCACTCCAAGTTCGCACCCCCGAAGGTGGCTGGCAAAGATGATATTACTGGAGAGCCTTTGATTCAGCGGAAGGATGACACCGCGGAGGTTCTGAAGAAGAGGCTCTCTTCGTTCCATGAACAAACCGCACCTTTGGATGCTGCTCTTGAGAAGCAGGGTGTAAAGATCGACAAGGTGTTGAACTTTGAGATTGACGATTCCATTCTGGAGGAGCGGATTACAGGCCGGTGGATCCACCCTGACAGTGGCCGTTCTTACCACTCCAAGTTCGCACCCCCGAAGGTGGCTGGCAAAGATGATATTACTGGAGAGCCTTTGATTCAGCGGAAGGATGACACCGCGGAGGTTCTGAAGAAGAGGCTCTCTTCGTTCCATGAACAAACCGCACCTGTGATAGAATACTACTTGCAGAAGGGGGTTGTGAAGAACGTTGAAGCCGCCAAGGCAGCGAATGTCGTCTCCAACGACATCCGGAATGCCCTCGCTTGAGCGTCCTCCTCATGTCAGCTGCGCCAAGTTCTTGAGCTTGAGAAATCGCTCTATCTCTTTCTTCTTCCTCATTGCACTAGATTGATCATTCTTCCGTCCAAATCGTGCAAGCAAGCCATCCTATTTGTTGCCTCCTAACTGTTTCTGTCACTCTAGGAGTAGCTCTGTTATAGCCATCTAAGAAGTTTAAGTGTGCCTTGTTGAACAATTTGAATTGAATAGTTGAATTGAATAATTGACTGAGGATGTCACATTGGCATTTTTGGGGGTGCAAGTGATGACTTCGGAGTATCTCAATGATCACAATCGGGTCAAGGTTTTGGTTGTAAAACTATCAACAAATGTGGCCATTTATGGTTTCAC

>ARP9 MG020638

GGGGTTTTTCTAGGGTTTGGATTTGCGGTAGTCAGTTGAAGCACAGTCTTCTTGCTTCATCGCTTTGCCCTGTCTTCTCAGCTGGAACTTTTGCAACTCCGATTCCACAGCATTCAATCCTTCGCAGCGACAACTGAGGAATGCGATCTGAACGCAAAACGTTGCTCAATTCTTTTAAGGCCAATTTCGTGGAATATGGTCTCCAGTGCTTTGTTTTGTAGTTGTAGGGTTTAAGGGAAGGACTAGGCGATGGCCAACGCAAGCAGCGGAGGCGTCCCTCGCGGGGTACCGGTTCAGCCGACGAGCCAGCAGGATTATTACAAATCGTTGGTGCCGTCTCAGCTCGTTGCGGAGCGTGGCGAAGAAATTGTTGTCATTAACCCTGGCTCTGCAAATGTTCGAATTGGTCTTGCATCTGCAAAGGCTCCTGTGAGCGTGCCGCACTGCATTGCTCACCAATTGCGCATGAGTGGTAGTGAGCAAGATTCCGCCGAATTACCGAAGAAAGGAGCAATCGGCGATAGCCGGTTTGGAACGTCATCATTATCAGCATCAAGGGTGGATGAACGACAAGATGCCCTTGAGCTGGTGGTTTCGCAGCTTAAGGTCAGGTCATTCTTCTCTGATGCTAAGGCAGGTGCGGGGGATTCGAACAGTTGCGAATTGCTTTCAAAGGAGCGGGATACAGGTTTTGAATGGACTAATGTAGAGGAGAATTTTGCTGGGCGTTCAGTACCAAGAATCCAGAAATCTTCAATCGCCAATGACAGAACGGACGATTTTTACAAGAATCATGAGAGAAACAGCGGCAAGGTCACAACAGATGCAGATGACGCTAAGGATATGGAGCTTCATAAACAGAAGCTTGACAGGTTTAAATTGTATCGCAAATACATCTGTGGGGAGGAAGCGTTAAAGATTCCTGCAAGCCTGCCGTACACTCTATATCGGCCTATGTGTCGTGGTCGTCTCAATATATCTAGTAAATATTCTATGCAGCAGGTCTGTGATGATATCTACAGAATATGGGATTACATTTTAACAGAGAAGTGTCAAATTGGCTCCAAATTGCGACCAAGATTTTCAGCAGTTTTGGTTGTCCCCGATACTCTTGATAATCGAGAGGTAAAAGAGTTGCTGTCAGTGGTTTTGCGAGATCTTCAGTTCCACTCTGCTGTTGTAATTCAGGAATGTGTAGCGGCTACTTTCGGGAATGGATTCTCTTCAGGTTGCATCGTAAATATGGGTTCACAAGTTATTTCAGGCATGTGTGTTGAGGAAGGGGTTGCTATTCCATCAACACGTTTTTTGCTGCCTTATGGTGGAGATGACATCACTAGATGTTTATTGTGGATCCAACGGCGAAAGAAGACATGGCCTATTGCTGACACTGATCTGTTGCGTGATCCATTGGACTTTCAAACCCTCGAGAAGCTGAAGGAGACTCACTGCGTGCTTTTTGACGGCGAGCAGCACACTACAGTTGATGTCCGGTGCCGCATGGCAGGCGAGCCAACTCGAGTGTACACAATTTTATTGTCATCTCTGAACATTCCACCCATGGGGCTGTTTTACCCGTCTCTTCTTTCATTGGAGGAGTTTTCGCCCCTTTCGCGACCCTGGTTTCACATAGACCACGAAGATAATACAGACGATGCTTTTTCTGAAGCTGGTCGACGCCTGGAGACCAATGAAACAGGACTAACGAATGGAAGTCTTAATGGAAATTCTATTAATAACGATTCTGATCCGTACGACTTTGAAGACAAAGAGAAGAAAGAGGTGGAGGAAATTTCAAGTGGTCTTGCGCAAGCTATTGTCAAAAGTATACTGTCGTTAGGACGCGTGGATCTTCAGAAGAAGCTGTTTGCAAGTATCCAGCTGGTCGGTGGCGTTGGATTAACTAAAGGGCTGGTGGATGCTGTTGAGGAAAGGGTTCTCCATGCCATACCTGTGGATGAAGCTGTGGACACTGTGGAGGTCCTTCCTAATCGAATGGATCCAATGAATATAACGTGGAAAGGTGGTGCGGTTCTTGCTGTTTTGGACTTCGGACGTGACTCTTGGGTGCAGTACGAAGATTGGTTGGATGGCATGGTCATGGTTGGCAGTGGCAGGAAGTATAGGGACTCGAATACTCTTCAGGCTCAAGCATTTTGGTATAATGCCATGTTAGATTAAGCTTTTAGCGCTCGGACGTCCAATTTGTGGACGACGAACAGATTGATATTTTCGGATCTTTCTCATATCACTGTTTGGATGAACGTCATCCTAATTTGTGGTGATACTTTATCTAG

>E2.299 MG020639

CTTCGGCATTGAAGGAAGTCCTCAAGTGTTTATTTTGAGAATAAGAAAGTGGCGTCCGGGAGTGGCTCTTCGATTTTCAGAAGAGGAGGGGATTCATCGCTTGCAATGCTGGATGTATCGAGAGTTCAGAAAGAGCTCGTGGAGATCGAACGTGATAAGAAATTATCAGGAGTGAGCATTGAGATCTCGGAGGGAGACTTGACGCGGATGCGCGGCACCATCAATGGACCTGTCGGGACGCCTTACGAAGGTGGCATCTTCATCGTCGACATCCAATTGCCATCTGCCTATCCTTTTGAGCCACCGAAGATGCAGTTCGTTACAAAAGTCTGGAAGAGCTTGGTGAATAGCCTGGAGTTTACGGTTGGCAGGCACCCAAACGTGAGCAGCCAGAATGGCGCCATCTGTTTGGACATTTTGAAAGACCAGTGGAGTCCAGCTCTAACTCTGAAAACGGCCCTTCTTTCTCTTCAAGCACTTCTGTCAACCCCAGAGCCTGATGATCCTCAAGATGCAGTTGTCGCACAGCAGTATCTGCGAGACTATGCAACTTTTCAGGGTACTGCTCGGTACTGGACTGAGACATTTGCAAAGAGRGGGTCTCTTGGACTTCAGGAGAAGGTAGCCAAGYTGGTGGAGATGGGATTCTCGGAGGACACAGCGAAGGTTGCGCTGGAGAGCTGTGGGGGCGATGAAAACGCGGCGTTGGAGAAACTTTGCAGTGGTTAAAATGTGAAGTATTCTSAAGTATTTCTTCCATTTTTTTGGACCAATCTATACCAGTGATCTCACTCACATGCAATTTTGGTTGTAATTGCCAACACACACGGCATGCTCGTTTCTTYGGGCTTCAAGTTGTTTGTAACAACTYCTATGGGTTTTGAGAAGGGACTGCCATAGACAAACACTTTATTGTGTTAGCTCGTTTCAAGTGGAGATTTTGACGAACTTACTSTCCTTGATGAAGACTGCTGATTGAAAGYAAATSGTSGGCTGGTTGAATTGATGTTTGTCTAATTTTGGGCATTTTTGTATATAAA

>E3.528 MG020640

ATGCGCTTCAGGAGTATTTTGATTGAGGTTTCGGGGGCTGTTTCCGAGACGTTTTGCGTTTGGAGGTCAAACGAGGCGATTGCAGTCTTTGGAATCCGTTTGAGTAACAGTGACAAAGAGAGCTCAAAGGATTCTGCAATGGAGAGGAATAAAGAAGAGGCAGCCGCAAGCTCAACTACTGAGAAAGCGCAGCAATTAGAAGGTGCTGGAAATAATGCCGAAAATGATGCAGCCARTACTGATAGTCCAGCGCCGGAGCCAAGCACTGCGGCAAGAGTGCCATTTACATCTCTGAGCCAAGTCGATTCGGACATGGCTTTGGCTCGGGCCCTTCAGGAACAGGAAAGGGCTTATTTCTTACTTCAGATGGGGCATGGTGGCGGCAGTTTCAATGACACTGATAGATTTTAYTATGGGGGAGAGCATCTGGGTATTCCTGGTGATGATGAGGAAGACCAAGATGACGATGAGGACGATGAGGAAGATGAGGACGAAGGAGAGGCAGAGGAAGAGGAAGAAGAGGTGAAGGATGGTGAAGAGGATTTAGAGGAAGCAGGGATTGAGGCAAGGACCGCTACTGATGATCATATCGACGGTGCCAATTTTGATAGTGATGAAGCTTTTGCTCGGGCTCTTCAGGACAAGGAGGACAGGGATACCACAGCACGTCTAATGGCACTCGCTGGTATTAATGATCTTGACGGGGAATTTGAGACCGACAGCAATGACTCTCAAGAGGGCATGTGGGAGGATGTTGATCCAGACAACATGTCTTATGAGGAGCTAATTGCACTTGGAGAAGCAGTAGGAACCGAGAGCAAAGGCCTCAATCCTCAATCWCTTGCCGCACTTCAACAATTCACATTCGTGTCTGATCCGAAACACACCACCTCTGATCAAGAGCAGTGTGTAGTATGTCGGATTGAGTATGAGGAAGGGGATAAGATGGTGAGGTTGCCTTGCAAACATCAGTATCACTCCGCGTGCATTCAACAGTGGTTRGAACGTAACAAGGTCTGCCCAGTCTGCAGTGCTGARGTTCCCTCTGATTCTAGTACATCTGAAACCAAGAGTTGAGTCGTTTGCTGCAAAGATTTAGTTCAATSTTTTGAAGGCTTTGCAAGTTGGCTGCTTTCATTTTATCATTTTTTTTAATAGAAATTCAATCTCACCATGTTTTATTTCTGTTGTAGCACACTATCAGTAGCTTCTGGTGAGCGCTTAAAGTGTTGATAACGATACCCATTATGTATCTCAATTTGTAAATTCTTCCTTCACATCTGGATGGGGCWTAAATTTTC

>EF1.278 MG020641

GACCTCCTTCACCGCCTCCGTCTCCAAGTCTCGGTCGCTATGGGTGCCTCATTCGAGAATCTGTCCACGCCTGCTGGCTTGAAGAAGCTCGACCAGCACCTTGCTACTCGCAGCTACATCTCAGGGTACCAGGCTTCCAGGGATGACCTTGCTGTGTTCGTCGCTTTGGAGAGCGTACCTGCGGAGTACGTGAACGCTGCGAGGTGGTTCAAGCATATCTCAGCTTTGGCTGGACCACAGTTCGCTGCTCCAGGAGTTGGAGTGCAGATCGAGAGTGGTGCTACCCCTGCCGCGGCCCCGAAGTCGGTGGAGGCTGTCGAGGAGGTCTCCCCGCCTGCTGCGGATGCTGATGAGGACGATGACGACCTTGACCTTTTCGGCGACGAAACTGAAGAGGAGAAGGCCGCATCAGAGAAGCGTGAGGCCGAGAAGAAGAAATCCACCAAGCCTAAAGTTGTTGGAAAGTCATCTATCGTTATGGACGTMAAGCCTTGGGATGATGAGACCGACATGGTGAAATTGGAGGAGTGTGTTCGCGCTATCCAGATGGAGGGTCTTCACTGGGGAGCTTCCAAATTTGTGACTGTTGTTGCTGGTATCAAGAAGCTGTCGATCATGATGACCATCGTGGACGACTTGGTCAGTATCGACAACCTGATTGAGGACCACCTGACTTCAGAGCCCAACAACGAGTATATTCAGAAGACGAGTCCTACCAATGCTGCCAGTGTTACGATTCGTATCTATGATATCACAATGAGCTTTCAACTGGGAACGAAGCCTACTATCTAAGCCCTGGACGCTTCTTCTTTCTGGGATACATTTTTGAATCTTTCTTTTATAAAGCGCTGTTCTTTTTATGTTTCGACTTCAATTGTCCTTGGCTGCAAGAGCTGGTTGTATGTGTGTTCAACCTTGCACTGATTCTTCGAAGAGTGTCGAATTCGGACTCAAATATGTTAGCCCTTCGGAAGTCTTCGGAATTCGTTAGTTCTCTTGACAATCTGCCGTCTAGGTCCGAGTAGCTTATGAACCTGATGAAATCCATTTCAAATTTGCAGACTGGTGCGTCCTCATAGAATGGTCGGTCTTGTTTGTCTACCTTATCCAGAGCCAGAAAAGTGAAACCGTCAAGATATCAATGGATCATATCAATCCGTACCTCAAATACTTCCCGTAGTCAATTTCAAGATGCAGACTACTTCGTAACTATGGAAAKATCACCCATCGCCAACCAGGGATTCATACCCAACCTGTAGGTATAGCAGCCACTAGAAAACCCTGCATTCAGAAAACTACTATTGATGATATATATTCATAGTCCTGAAAAGTAAACAAGTAGAGGATACTATGAATTTGACGGGTAATCTGTTTCAGCGAAGTAATGCGGACACATCGAATTTTTTGCATTAGCAAACTTTAACGCATACTCGGATTGTTGCGGCCTTGCCAAGGATGAGTATGGAAGAGGGACCTGTAACCAATTAAGCGATTATGGACATGACCTAGGGTAGTGTTATGCAAAGTAAATATTTTCAGAGGTGTATTGACTGTAATGATGGAAATTGCAAAAACTTTGG

>GAPDH MG020642

TGCCTTGCACCTCTCGCAAAGGTTATCAATGACAAGTTTGGAATCTTGGAAGGACTCATGACCACAGTGCATGCCACCACAGCCACACAGAAGACCGTCGATGGACCCTCGCACAAGGATTGGCGAGGTGGGCGTGCTGCCAACACCAATATCATCCCGAGTAGTACTGGAGCTGCCAAGGCTGTGGGCAAGGTGTTGCCAGAGCTGAATGGGAAGCTCACGGGAATGGCCTTTCGTGTTCCGACTACTGATGTGTCGGTGGTGGATCTCACTGTTAGGCTTGAGAAGCCTGCTTCCTACGATGCCATCAAGGCCGCTATCAAGGAGGCATCTGAGGGGAAGATGAAAGGCATTTTGGGTTACACTGAGGACGATGTGGTGTCCACGGACTTCATTACTGACAGTAGGTCTAGCATCTTCGATGCCAAGGCTGGAATTGCTCTCAGTGATACCTTCGTGAAGCTTGTTTCGTGGTACGACAACGAATGGGGTTACAGTAACCGTGTGGTGGACCTGATTTTGCACATTGCCGCCGTTCAACATGGAAGCGCCTAAACAAATAAGTCGTTGAAAGGGAACTCTTCGCCGTCCTTTTATTAGTTGATATCTGTTGGGACCAGTTTCAGTGCACAAACGGTGTAGATATTGACCAGTCAGTCATTGGTCATTTTTTTTGTGATGGAGACTTTTGATCTGTCATTGGATCTCACGTGTTACTCACTTACATGTAGCTCAACTATCGGTTGAAAATAATTTTGAGATCCGTTTGGTCTTGCTCTTCGAGTAACTGTCTGAAACCAAAGACTGCATTTTGAGTTGTTCTTTTTTTGGTTTTTTGTCTAAATGAACTAGACCTGCAACCTTGGAATATGCTGGCCTTGCAAATCTCAAGAGAAAAGTAGTTGTCATTTTCTGGTTTGAGTTGATCCATCACATCGATTGAGGTCATTTCTGTAGAACAGAGTTAGAAGACGATTAATCGTTGCATACTGAGGGTATGATTATGGCTTTTACTCTTTTCCTTCTTTACCGTTATAGCGTTCTATAACTTTGATTTAGCTGAGGA

>HMT MG020643

TCCCTTTGCCCATGTGTCAGGCCCCTCCCCCTGAAGGATTGGCAGCGGCGGATGTTGAAGAGCCTCCTCCTGCTGACACACCAAATAAGTCGGACACACCCAACAATGCTGGAGGCAAGCAAAGAATGGCAGCCGAACGAGGAAACGGCACCGTCGAAGCCCGTGTTTCTGTTGGTAGTGGTGTACGGACTAAACGGGCAACTGATGATAAAGATCGGGGCAAACGACCAGGGTTGCCAGTAAAGAAATTGTCCCTTCCAAAAGGCCCCAAAAAGATAAGTATGCTTCCTCGAACGAACCCAGTTGCCAAGTCTCCTATGAGCGATGCCTCGCTGCACATTGAATTGAATGAGGGCTGCAGTAATGACGGGAGTGGAGACTTCGGAGTTGGGCCCTCCTTGCACGACCAAGGCAATAGCTCGGACACACAAACAGATTAAGGAATGCCATTCTTCAAAAATCCATTGAATGGGCATGCTGGGCGAAGGTCAGACGTGAGGTCAGACGTGAGGTCAGACTCTGTATGATACCATGAAGAGGGTGGATCCAACAGATATTGATGACTAGGGATGCCGAGTCATACCTTCCTCGGTGTCGATGTTCTTGAATTTGGCTTCATTGTCTCAGCACATTTTTTGGGTTTAAATCTCGATAGGAGTCCTGAAATCAGGATTTGCAAATTCAGGTTATTGTAGAGGATGTGTACCTGAGAAACTCGACCAATTTCTCAAATTTTCTCTTTTGGAGGGCATTCAAGCTCAAATTACAGCATTTGGTTGTGGTGAGGGTCCAGGTTGTGGATCTGTTGATTCGATTCTTCTAGTAAGGTTTGTGGTTGAAGGCAGTGTACATTACAAGGTAGCCTTCAGATGAGTGATTTACATGGGATCAGTCATATTATGGACTGGTTTGCTGATCTACTGTAGATATTTTCATTTGCGTGTTTCCGTTTGATTTTCACAAGGTTTGACCCTACTCAACTTCGTAACTTCTCTACATTCTATCTAATCAC

>HIS MG020644

TTACCCTTTCGTTTCTTCTCAGCCTTGTTTAGTATACTGTATGAGTGTCGCACCACTCGCACGTTCTTGAGGAGAGGCCTTTGGTGTAAGTTATCTTTGCCTGCGATCATCACTATTCTACATCAAACAAGAACTCATTATCCTGTATACAGAATGGCTCGTACTAAGCAAACTGCTCGGAAGTCAACCGGAGGAAAGGCTCCAAGAAAGCAATTGGCCACCAAGGCTGCCAGGAAGTCTGCTCCTACCACCGGAGGAGTCAAGAAACCCCATCGGTATCGCCCAGGAACRGTTGCTCTGCGTGAGATCCGCAAGTACCAGAAGAGCACAGAGCTGTTGATTCGGAAGCTGCCATTTCAACGTTTGGTCCGTGAAATTGCCCARGATTTCAAGACTGATTTGCGCTTCCAAAGCCATGCTGTSCTTGCCCTCCAAGAAGCTGCRGAAGCTTACCTCGTGGGTCTCTTTGAGGACACCAACTTGTGTGCCATCCATGCCAAGAGGGTGACCATCATGCCCAAGGATATTCAGCTTGCCAGAAGGATCAGAGGAGAGAGAGCTTAAATAGGCTTTATACATGTCAACATATCCGAAGCTAGTTTTCGCAGAATGTCACCTCTTTGGGACATTGTAACAGACATTATTTAGTGGCATGCATTCCATGTTATTGCAATCTACAAGAGAACTCTATTCAAATAAAG

>POB1 MG020645

ATGGCGGACCCTGGTCCCATCTTCCTTGCCCTTGTTTCCCTCCTTGTGACCACCTCTTATTATCCCTCGCCTCATTGCGCTCTCTCATTCCCGGTTCGAAGCGGTGTCGGAGATCGCAAGCAGGTCGAGATAGGGACTGCCTATATGGATCATGAGGGTTGCAGCAGTGGGGGCATGTCAGCCCCGACCACRTACACATTCGCATTYAATGACAGCAACTTTTCGGATCGGCTTCTCCGGATCGAGGTCGTGGCTGCATCAGAGAAGAATGATGCAAATGGTACGAGTGCTAGGCARAAGAAACGGCGACGTGCTGACCGCAATACTGAAGCAGTCGCTGAGGGTGTTGCGAAGTTGTTAGGCGATCATCCGGAAACTCAATTAGTAGAGGGTCAGGAGGAGCAGGTTATGTTTGTCGGTGACGATGTGGCACCTCAAGAAGCGGATGAAGAAGCCGTAGCCATGATGGAAGAGCCATATGGAGTAAACAGCATGTTCATGGCACATGGAGGGGAGGACGGCGGGACTTCGTCTGGATCTTGGAACATGGATACAGCTGTTGTTCTTCGGAGCAAGACGGTACATATCAGCTCGGCCATTTTGGCTGCYAAAAGCCCCTACTTTTATAAGCTTTTTTCTAATGGGATGCGGGAGTCGGAGCAACGCGATGTCACTCTTCGGATCATCCAATCTGAGGAAGCRCCATTGATGGACCTRCTCCAGTTTATGTACAGTGCTAGGGTTCAGGCAAACACTCCATCCACTGTACTAGATGTCCTAATGGCAGCTGATAAATATGAGGTTGCAACATGTATGCGCCACTGTAGCCGACTGCTCAGGAACCTGCCCATGACATCGGAGTCTGCTTTACTCTACCTAGACCTTCCGTCCAGTATTCTTCTTGCAGAGGCTGTTCAGCCTCTAACGGACGCAGCCCGCACCTACTTGGCAACTCGCTACAAAGACATYACTCGATTCATTGATGATGTCATGGGCTTACCTCTCGCTGGAGTTGAAGCTGTTCTCGCAAGTGATGATCTACAGGTTGCCAGCGAGGATGCAGTMTATGATTTTGCCTTACGTTGGTCACGTCATCATTATTACAAACTAGAGGACCGCCGGGAAGTCTTAGGCTCGCGGCTTGTTTGGCTGATCAGGTTCCCTATGATGTCCAGCAGRAAACTTCGTAAGGTGTTGACCTGCTCTGATTTTGAACACGAGTTGGCATCCAGGTTGGTCCTTGAGGCTCTGTTTTTCAAGGCTGAGCCTGCCCACCGGCAAAGGCAGCTGGCCATGGAGGAGACTATGCATAAACGCTACTGTGAGCGGGCATACAAGTACAGGCCAGTGAAAGTCGTGGATTTTGACACGTCATGTCAATGCCTAGTATATTTGGACTTGAAGATTGACGAATGCCGTGCCCTTTATCCTCAAGGGCGAGTCTATTCTCAAGCTTTTCATTTGGGAGGCCAGGGATTCTTTTTGTCCGCTCATTGTAATTTGGATCAGCAGGGTCAATGCAAATGCTTTGGACTCTTCCTTGGRATGCAGGAAAAGGGTTCCGTGAGCTTTGCTGTAGACTACGAGTTTGCGGCAAGAATGAAGCCCASCTGGGAGTTYACTCCCAAGTCCAAAGGGAGTTATGTATTTACTGGTGGCAAGGCTGTAGGRTACCGGAACCTGTTTGGAATGCAGTGGCAAGACTTCATTGCTGAGGACTCCCCCTATTTTCGTGATAGTATTGTTCATCTCCGCGCGGAGCTTACCATCAAGAAGCCGTCGAGCGGATGAATTTTCTCAAAATGGTTTGGAGGAACTGTACATTTCCAGATGTAAAAAATAAGCAACTCGAAAAATCAAAAGCGTAGATTAGGTTTCCAGTTCTGGTGACCATCCCAGAGTGATTAGCAGCCTTTGCTTTCCGTTTGAGACCCTCGCTGTTTTTAGCCTTGACACTTTGCGCAGCTTCGTAGATTATTGCTAGTCTGTGTGTGCAAAACCGAGTATTCTTAGGGGGTGTAACTTACTCTGGAGTTCGCATGGAATTATCAAACGGTTTTCCTGGCAATGACTCTTCCGGTTTGAAATGACTCTTTTGAATATACGTGGTCATGGGATTATTC

>SPT MG020646

ATGACGGTGGCCGAGCTGAATCCCGAGATGGAGAAYGAGGAAGGGTTTGAGGTGCCTTACCATGTGGCGCTCACCACGTATTTCTCGTATGCGATTCTGTTCATCATCGGCAGTATCCGGGACTTTTACCGCACTCTTCGCAGAACTGGGAAAAAAGGTTATGCTCCTCTCTGCAAGGATTTCGAGGATTTCTACACGCGCAGGTTGTATCACCGCATTCAGGATTGCTTCAATCGACCAATTGCGAGTGCACCCACCAAYTGGATCGATGTAGTGGAACGTGTGTCCTATGACAGCAACAAAACGCTGCACCAAACAGAGAACACAGTACGATGTTTGAATTTGGGTTCGTACAATTACTTAGGGTTTGCTTCTCATGATGAGTATTGTACACCGCGCGTCGTTGAAGCGATGAACAAGTATGGGGCCAGCGCTTGCTCAGCTCGTATTGACAGCGGCACTACTGTCCTTCACAACGAGCTTGAGGTGTTGGTTGCWAAGTATGTGGGAAAGCCAGCGGCTATGGTTCATGGAATGGGATATGCAACGAACTCGACCTCGCTACCTGTCCTAGTTGGCAAGGGCGGATTGATAGTTAGTGATGCTCTGAATCATGCATCCATTGTAAACGGTTCACGCGGCTCTGGTGCCAAAATTAAGGTCTTCCAGCATAACACAACATTTCTCCTGAGCGTACACGAAATGAAGCTCGAGGACTCAAGGGACTATTTGTATTTTCTAATAGACGCGTCCCATTTGGAGCAAGTACTTCGGGAAGCCATTTCTCAGGGACAGCCTCGAACTCATAGGCCATGGAAGAAGATCCTCGTTGTTATTGAAGGTATTTATAGCATGGAAGGCGAAACATGCCGACTTAAGGAGATAGTGACCGTAGCAAAGAAATATAAGACTTTCATTTATCTAGACGAAGCTCACAGCATAGGAGCAATTGGGAAAACGGGGAAGGGCATTTGTGAGCTACAGGGTGTAGACACTGCCGACATTGATGTGATGATGGGCACCTTCACCAAATCTTTTGGCTCGTGCGGCGGGTACATTGCAGGGTCTGAGGAGTTAATAAAGTACATGAAGTACACCAATCCTGCACACTTGTACGCCACTTCTATGTCAGTACCAGCTGTACARCAGGTTATTTCTGCTCTGGAAGTTATCCTTGGGCTAGACGGGAGCAATCGGGGGGCGATGAAGCTTACGCAGGTGCGAGAGAACAGCAATTATGTGAGAAGTGAGCTTCGAAACAGAGGCTTTGAAGTATTAGGTGACACGGATTCGCCTGTCATGCCCGTCATGCTCTACAACCCTGGAAAGATTCCAGCTTTTTCACGCGAGTGCCTGAAGCGCAATGTCGCAGTGGTTGTGGTAGCGTTTCCAGCTACACCCTTGCTGCTCGCAAGAACACGGGTTTGCATATCTGCTTCTCACACACGGGAGGATCTCGACAAAGCAGTGAAGGTCATTTCTGAGGTGGGTGAGATGTGCAACATCAAGTACTTCCCCAGGAAATTAGCAGAAGATGAAAAGTTCTTCTGGTACGATCAGAAGAAACTGGAATAGAGAGCACWAAACTTGCAAATCTCGGCAGTAGAATCTCTGTCCGAGTCTTCTACTGCTGGGCTTGTTCAACCAATTTTTTGAAGTCACTGGAGCTGCCGACACTGGAGAGCTTGAGACGCCCTACAGGATTTGAAGTGAAACGTTTCAGGTTTTGTTGGAGTACGGCGGGGTTTTCGGCGGGCGCTGTCTCTCTCTCGACAGTGAGCAAAGGCATAGGAATCCATTCTTTTACTGCGTATACTTCCTTGGATTGTCTCCCTCTCCTCTCTCTCCAGTCGTATTGGGTGTTTTCGCATGTTCTCGCACGAACTGGCCCTTCGGGTCTCCATTTGTAAATTTTAGTGCAACATGACATGAAACACAGTTAATTATATGCCTTCCACGTGTGAGGTCTCCAATTGATGTCATTGTTTGATGCAGTCATGGYAGAAAT

>tIF MG020647

GTAAAATTGTCAAGGAATTTGGGTCTCCTGAATTGTGCACTGGACGGTTTTCTCTTTGCGGTATTGACGCAAATAGCTGGTAGTTTTTGCATCTTCTTCATTTTTCGGTTTGTAAAACATTTTTCAGGGATCTTCATAACCTATTTTCTTTCTCTTGTCGGAGCTTTGCACTGGCGCTTCGTTGTCGGTAATCATCAATCTTCGTTCGCTGCGGACGATTTGCAGCAACATCCTTGATTTTTGCTCTTTCTTTTCATTTCCTCTTCTCGATTTTGTCATTCGGTTTTAGGGTTTCGGGAATTCAGGGCTCCGGGTAAGGAGATCTTTAGGATTTTCTGCACTAGTTTTCGCGACTTGTATTTTTGAGAGAATTCGAAAATTAGGTCTTGGGACTASAGAGTAYAAGAGTGGTTTTGTGATTGTTTGTGACCTTTTTGGTGCACCAGCATTTGGAAYAGACRATGGGGTTGCCGGCCATGAACTTCCAGGTGGTAGTGCTGGCGGGCGGGTTGTCGAAGAAGCTGTACCCGCTGGTATCCAAAGATGTGCCCAAGGCTTTGCTCCCGCTCGGCAACAAGCCCGTGCTCTCGTACGTTCTGGAGCTTCTGGAGGCCAGCAACTTGAAGGACATTATCCTCGTCGCTGCCGGCGAGGACGCCGCGCTCTGCGTCGGAAATTGGGTTGCGGACGCCGTTCATGATCGCCTCCGTGTTGAGGTTGCTGCAGCCCCTGAGGACTCGGACACCGCCGATGCCTTGAGGTCCGTGATGCACCGCTTGACTGCAGAGGACTTCTTGGTGGTGAGTGGGGATCTTGTCTCCGATGTGCCCATCGGCGCAGTTGCAGCTGCTCACCGACGCCAGGGAGCTCTGGTCACAGCATTGCTGTGCAACCGTGCGTCACTRGGCTCTTCGGAACCGGGAAGCGAGAAGATTAAGCAACAGCCTGTTAGTGATATCATTGGCTTGGACTCTACGCACCAGCATCTTCTCTATATGGCTCCCGGCGCAGAGATTGAGAGGGATTTGCGAATTCGTCGCAGCCTACTTCGCGCAGTTGGAAATATGGAGATTCGAACAGACCTTGTGGACGCCCATCTGTACGCATTCAACAGGCTCTTAGTGCAGGGAGTTTTGGAGTCGAGGCCAACCATTAAGAGCATCAAGCAAGATTTGGTGCCRTATCTGGTSAGGTCTCAACTACGATTRGGAGTTCCCTCAACAGCAGTGGGATCTCCTGTGGAGGATAAGGACGCACGGCACCAACCTTCACTCCCGGATACGGAGGTTTTTGCTCAATTACTGCGATCTTCTCAAAGTGGTCACCGTATCACGGGTTCYCCCCTCAAATGCTGCAGTTACATTGCAAGCAAAGGGAAACTCTGCGTGCGCGTCAATTCCTTGCAGGCMTATCTGGACATGAACAGAGAGGTTGCAGGAGAAGCCATCCACTTGACAGGCTACGAAGTCTCCGCTCACAACAATGTCATTCACGAAACGTCCTCTTTGGGGTGGAAGTCCACTGTTGGCCCTCAGTGCATGTTGGGAGAGGGGTCCACCCTTGGGGAGAAGTGCAGTGTGAAAAAGTCGGTGGTCGGCAGACACTGTCGCATTGGTTCCAATGTGAAAATCATCAACTCGGTGGTCATGAATTACGTTACTGTAGAAGATGGATGTACCATTCAGAACTCCATTATCTGCAGTAACGTGAACCTGCAAGAACGATGCTCTTTGAAAGACTGTCAGGTGGGTCCTGGCTATGTTGTGGGTGCTCGGTTAGAACTCAAGGGGGAGGCACTTGCAAAGAAGGAGAAGTCTTGAGCGCGGSAATTTTTTTTCAAAGGRTTGGGTTCCTCGGTTCATGTTCCATCTTAGTGGCTGTGTAAAGGACGTCCCTCCACTTTCGGAATTTCTATTGGTCATGTCTCTGGCCTTTCAAAGGAAGAGTGTACAGTACAGTATTAATACAGGGCTACCACCTTGGTACAATTTGAGCTATCATTAAGATTAGGGTTCAAGCAATCAGTTGCAACGTTGTCTTACGAAGTGATGTGTTCTGGACTTTTGATCCAGGCATCCAATTGGGAAAAAGTTGTGTTCCCACCAAGAGGATAAATGTTCACA

>TUB250 MG020648

CGGTGTTTTTTGAGGGGAGCGATTCACGCTGCTGCAGCTCTGTTTCCGAGCTTCTTGAAGTGCAGAACGACTTAGTTTCTTTTGTTTTTAGAGAAACACAATCGTTGTAGACAGGCGTCCGCTGAAGAGGTTGCAAGATGCCTCGCGAAATTATAACCCTACAGGTGGGACAATGCGGGAATCAGATAGGAATGGAGTTTTGGAAGCAGCTGTGCTTGGAGCACGGCATTAGCAAGGACGGCATTCTGGAGGATTTCGCTACTCAGGGTGGCGATCGCAAGGATGTGTTCTTCTACCAGGCGGACGATGAGCATTACATTCCGAGGGCGCTGCTTATTGATTTGGAGCCCCGCGTAATTAACAGTATTCAGAATAGTGAGTACCGGAATTTGTACAATCACGAGAACGTGTTTGTGGCTGATCATGGAGGAGGAGCTGGAAACAACTGGGCGAGCGGGTACCATCAGGGAGAACAAGTGGAGGAAGATATCATGGACATGATAGACAGGGAGGCTGATGGAAGTGACAGCCTCGAGGGCTTTGTTCTTTGCCATTCTATTGCGGGAGGAACGGGTTCAGGAATGGGTTCGTACCTTCTTGAAGCGCTCAACGACCGATACAGCAAGAAATTGGTCCAAACTTACAGTGTCTTTCCAAATCAGATGGAGACTAGTGATGTGGTTGTCCAACCCTACAATTCTTTGCTTACGCTGAAGCGCTTGACCTTGAATGCGGACTGTGTCGTGGTTTTAGACAACACGGCCTTGAATCGTATTGCTGTGGACCGGCTTCACATCCCAAATCCGACGTTTGCGCAGACTAATTCCCTGGTGTCCACAGTTATGTCAGCCAGTACCACAACTCTCCGTTACCCAGGGTACATGAACAATGACTTGGTTGGATTGGTGGCTTCCTTGATACCTACTCCACGGTGTCATTTTCTGATGACAGGGTACACTCCACTGACTGTAGAGCGACAGGCTAATGCCATTCGGAAGACAACTGTTCTGGATGCCAAAAATATCATGGTTTCATCATACGCAAGGACAAAGGAGGCMAGCCAGGCAAAGTATATCTCCATACTGAACATTATTCAGGGCGAAGTTGATCCAACTCAGGTTCATAAAAGCCTACAAAGAATACGAGAAAGAAAGCTAGCCAATTTCATCGAGTGGGGACCAGCAAGTATCCAGGTCGCGTTATCACGGAAGTCACCGTATGTTCAAACTGCACACAGGGTTAGTGGACTTATGTTGGCGAGCCAYACCAGTATTCGTCATCTTTTCAGCAAATGTATCAGTCAATATGAGAAGCTAAGAAAGAAGCAAGCGTTTTTGGATAACTATCGCAAGTTTCCTATGTTTGCTGATAACGACCTGTCCGAGTTCGATGAATCGAGAGAAATTGTTCAAAACCTAGTGGACGAGTACAAAGCTTGTGAATCTGCTGATTACATCAAGTGGGGCATGGAGGATCGAGGAAAGCAGGTGTCCGGCGAAGGAAATACGAGCGGGACCGTGGATTCCAGAGTAGCCCCCTCATAGAATATTTATTTTCTAGCGCCTAATTTTTTGTAGTCATACCAAGTCCAATGTGCAGTAGTCGTGTTTTTTCTTAATCTTCAATTTTTTTTTACCATTTTCTACGAAGCTACCATTTTGTGACCAGTTMCTGGCATTGCCGACTGAGGTCAGTCCGTCCAGAACCTTTGGCCTTGGTCACCAATCATTTGCCCTAAGCTTAGTTGTTGACGAGGTGACCATGACGTTCCTTTAACGACTCTGGCATCAGGGGAAGCGTGCTGTGTGTTGACAATGTAGCCTCCATTGAAAATCCTGGATCGATGGAAGACTTGCGCTCTTGAACTCACATCAATATTGGAGCCTCGTGGGCATGTTTGTCACTTTGTCTCTGAGGCGAGGAATTTCTAGGCTCAATATTTCAACTGCGAAAAAG

>UFD2 MG020649

ATGGCACAGCCAAAGGCGGGGAGGACGTTAGCAGATCTGGAAGATGCAGTGCTCCGCCGCGTGTTGYTGGTGACAGTTCGAGCGGGTAGCCAAGAGGGCCTGGGTTTGCCGATTTATTTGGAGCAGCTCTCTGCAGAGCTTCTGAGCGAAGGGCGTCCCATGCTTCTCTCGCGCGATCTCCTGGAGCGCGTGCTGATGGATAGGTTAAGTACTTACAATGAGGGCATGGAGGCACCCTTTCTCTACCTCGTGAATTGTTACCGGCGCGCTTTTGACGAAAGCCGGAAAGCACARACCATGAGGGATAAAGCAGGTCTTGCTGTGGTGAACGACGCGTTGCARCAAGTGAAAGATTTGACAGTGTCGTACTCTGTGCTCATGCTAGTGCATGCCAAGTATGGCATGTTTCCTCAGCCTCTGGACGTGGCTCATCTYGGTCCTGATGCGTTGTTACTGGCGTCGCTGATGGCGGATGGATCCTCCAGCGCAGGGTTTTATGCCACAAGCAGTGGTGTGGAGCCGCTGCCGTCCGGKTTTTTTGAGCAATTGCTGAAGCGRTTTGAGGATGAACCGGAAGGRTTCAAGTTCGTCTTTGAGCATGTATTTAAGGATTTGCAAACGGCTATGACGAARGTGTCTCCGTTGGGCCCCTTTTTACCATGTGTGCGGACTTTGCTTATGTTGGTGTCGAATCCTCCCTTAGCGAAAGTGTTGGTGGAGCATCCAATGTGGAACCCTAAAGGGGCATATGTCAGTGGACGGACTTTAGAAATAAACAGCATTCTTGGCCCCTTCTTTCACATYAGCGTTTTGCCAGATATTTTACCTTCCGGAGAGCCTAACGTGAGACAACAATGTTTCTCTGATGTGGCGAGCCGCCGACAAGCTGATTTAATGTCTTCATTTGCCACTATAAAAACGGTCATGCATCAGCTTTACGATGGTTTACACGAGGTGATACTGAAGCTGCTTCGGACACCTGAGACACGGGAATCTATGCTCCAATACTTRGGGGATGTTATCCAGAAGAATGCTAACCGTAGCCAACTTCAGGCAAATCCGTTATATGTTGCGAGCAGTGGAATGTTCGTGAACTTGAGCGCAGTCATGCTCAAACTTTGCGAACCGTTCCTGGATGCTTCTTCAAGCAAGAGAGATAAACTTGATGCTCGATACGTTCTTCAAGGAGGTCGGCTGGACTTCAGTGGATTGACGACAATCCTCGCCACTTCAGAGGAGTTGGCGAAATGGGTGGACAGTCGCAATCATGCTCGTACTGAAGGTTTTCGCCAGGTACAGCAACTTCGAGAGCAAGAAGAAATGCGACGTTTACAAACGGAGGAAGCTTCTACTTCAATGACAGATAGTTTGCAATCGTATCCACTGAAGTCCATGGCGTCTGCATCGTCGGACAATGTGAAGTTTACGTTCATCTGCGAATGCTTCTTTCTCACAGCAAGAGTACTCAATTTGGGACTTGTTAAGGCACTGTCGGAATTTAAATCTTTGATGCAAGAACTSTCTAGGCGGAAGGAAGAACTCACGACATTGAAGAACATGCGTGGTGATGGAGCACCACCCCAGTTAGAACAAGACATTTCCCAATTGGAGGCAAACGTTGAGCAACTATCCCAGGATCGTCTGTGTTATGATTCCCAGCTCCTCAAAGACGTTGATCTGCTGCAAGAAGCTTTGGCTTACTACAGGCTTATGATAGTGTGGCTCGCTGGTCTAATTGGTGGGTTTCGTATGCCGCTTCCAGCTCCTTGCCCTATGGAGTTTGCCTCCATGCCCGAGCACTTTGTGGAAGACGCCATGGAGTTACTCCTGTTTGCATCTCGCATTCCGAGGGCTCTCGATGGTGTGAATTTGGATGAGTTCATGAGYTTCATTGTCATGTTTATGGGGAGTCCACTTCACATAAAGAATCCGTATTTACGAGCTAAGATGGTGGAAGTGTTAAGCGCATGGATGCCTTCAAAATGTATTTCTCCAACCTTGAGCAGCAGCATGTCATCTCTTTTTGAAGGACATCAGCTGGCTTTGCAGTATTTGGTGCCGAATCTTCTCCGACTTTACACGGACATTGAGTTTACTGGAGCTCATAATCAGTTTTACGATAAATTTAATATTCGTCACAACATTGCGGAGCTGCTGGAGTATTTGTGGGGTGTACCTAGTCATCACAACTCTTGGAAGCAGGTTGCCGCTAAAGAGGAGAAGGGTGCGTATTTGAGGTTCCTGAATCTACTGATTAACGACAGTATCTTTCTCCTGGATGAAAGCCTGAAGAAAATACCGGAACTCAAGGAAATGGAAGCGCAACTGGCTGACACATCTGTATGGAGTAGACGACCTGCTCAAGAAAGGCAGGAGAGGGAGCGCCATTATCATCAACAGGAACATGCGGTTCGGATAGACATGATGTTGGCAAATGAGGATGTAAAAATGATTCAGTATACATCTGCTGAGATCACAGCACCATTCTTACTTCCTGAAATGGTTGAGAGGATAGCGGCCATGTTAAACTATTTCTTGTTGCAACTAGTCGGACCTCAACGCAAGACCCTGAGGTTGAAAGATCCTGAGAAATACGAATTCAGGCCTAAGGAGCTGCTAGCACAAATTGTAAATATATACGTGAACCTGGATAGGGGCGATTCTCAAGGGATCTTTGCAAGAGCAATTTCCAGCGACGGTCGCTCATACAGGGATGAGTTGTTTACAGAAGCTCTTGGAGTTCTCAAAAATCTTGGTGTCCTGARTATGCAAATGCTTGAAGATTTTGAAGCACTTGGTGCTAAAACGAGAGCGAGTTCACAAGAAATGATGGATGAGGAAGCTTTGCTTGGTGATATTCCTGAAGAATTTCTTGACCCTATTCAGTACACATTGATGACGGATCCAGTTATTCTTCCCTCATCGAAAACCACTGTTGACCGGTCTGTTATACAGAGGCATCTTCTAAGTGACCAGACGGACCCATTCAACCGCTCGCTCTTAACTGCTGACATGTTGATACCCGACYACGAGTTGAAGAGAAAAATTGACGAGTATCTTGCGAGTCACTCAAAGAAGTGACCTTTCCTACATTGACCCCATTTCAATTATGTCCCATTTCTTGAGTTGATATGCTGCAATGCCACGATCTGCGCTTTGGAACGTGGATAGTTCGAATGAGATAGGCGAGAAAATGGTCCCTGCACTTGTGAGGAGTTGCTTCCAGTTTTGAGTGGGAATATCTCAATTATTGAAATGAGTATTACCTACTTACGCACCGTTTTCTGTGCAGCATAGCAATCTGATCTTGCATCAAGAGTGGTTGCTAGATTATTGTTACTAC

>UK552 MG020650

ATGGCTCTCGCAGCGGTGGCCTGTGGCCATATGACTGTCACCCTGCAACCGACAGTCCTCGCACGCATGTCCAGCTCGTCGACGGCGCGCAATTTGACATGGAACCCTCTTGTGGTGAGGGCCACCAGTCGACGATCCGACATGAGCCAGTTCTTCGGCGCGGAATTGATACGTTGTCGTGGGGCAGCCCGGCTCAGTGGTGCCAATCAGAGGAGACTTGGCTGGGGCATCAAGGCGGCGAGCGGCGAGACAGTGAGCAAGGGCGCCCGCGCTAACTACTCGATGAGGACGAAGCAGCCATCAACGGAGGCGGAGGCCGAGCAAACGTGGTTGAAGCGAGGGTTCGAGGTTGTGGTGGAAAGTGTGAAGCGATTGTCGAGACCAACTATGGCGGTGATGCTAAGCCTGTTGCTTTCGCAGAGCAATCCGGATATGGCTAGCGCGGCCGGGGGAGGGAGAGTGGGGGGACGGGTAGGTGGGGGAAGCTCGTTCTCGAGCAAATCGTTTTCCGCTCCCAGCCGATCGTATTCAGGACCGTCAGGGGGGTACTCCACGCCCAGGCAGTACATTGCACCCAGCCCTGGATTTTCATACGCTGTACCGTACGCGGCTCCGTCGCCATTCTTTGGCGGTGGCCTGTATGCCGCGCCTGCTTACGGCATCGGCCTGGGCGGCGGAAGTATCTTCTTCCTGGTCATTCTGGGCTTCATCATATTGCAAGCCGTGTATGGCTTTGTGTCGGAGCGGTCGGGGCTAAGTGGGTCCTTGCTTAATGGTGCGCAGGTGGTCAGCGTTTTAAAGCTGCAGGTTGGGTTGTTGGGCATGGGTCGTACCCTCCAACGCGACTTGGACCGAATTGCCGGGCAAGCYGATACCACCACAGCCGAAGGTCTCCACTATGTTCTAACTGAGACGTGCTTGGCKTTGCTGAGACATCCTGATTATTGCATCTCTGGCGTCTCGTCGCATGACATCAGTCAGTCCTTAAGGGTGGCAGAGGAGCGATTTAACAATTTCTCTTTGGAGGAGAGAGGGAAATTTGATGAGGAGACTCTTGTTAATGTGAACAACTTGCGGAAGAGAATGATGGGAGCGCCTAAGTCTGAACGATTCAACAATGAGTACATCGTGGTGACGATCCTAGTAGCTGCCGAASGGGACTTGAAGCTTCCAGCGATCAATGGTAATGCTGATCTGAAAGCTTCTCTGAGGAAATTGGGCTCAATACCGGCAGACTCAATTCAGGCTGTTGAAGTATTGTGGACGCCACAAGACGAAAATGATACGTTGAGCGAAAGGGAGCTTCTCCGCGACTATCCACTTCTCAGATCTCTGTAGATCTGACGTAAGCGTTGGTTTGAACACGAACATGAATATAGATAATTAACCGTAGTACAATAGAATTGGAGCAAGATTTTTGATCCTCAGAACCTGTGCATAGAGTGAGTCTGGTGTTGGCGTTGTGTGAAGTAGGCGAAGAGAAATCGCAAGCTTTCGGACCCGCAATCGGAGAGTTGGGAGTTTTGGCCTTTTGTTGCCGGTGTGGCAATGCAATTTGAACATCTTCTGTAGCATTGTTACCTTGAGAAGATTTTCAAAATTGCCTCGCAAATTATGTGGATGAGAATGCGTGCAGCGTGGCGATCTGATTGCTTCGTGGATTGAAGTCACGAAGCTTTTATTGTGCGGTAGAAAACGAAGAAAGGACTCACTGGCTATGGCATGGCGATTATTAACTACAGTAGATGAGTTCCTATCACTAATATTATTACTGTCTTCGTCCCAAAATGATTGCTACAATCTCTGGTATTTTTGGGAGTCAATTTCATTGATCATATTATGAAAAACGTAGGTGGGTTTCAAATTTCATATCACCAGAATCAA

>SuTub MG020651

AAATGTGGAATCTAAGTTACCAGATGCGTTTTTAATTAGTGTAATCTTCGGATCGTAAGCGTGACTTTCGGTTGGTTTCCCTTGAAAGATGCTTTTTGAATTTGAATTTCAAGTCGGGGATACCGGCCAGAAGTGGACTCGGTCGCGGCCCATAGCCGGTGATGGAAAGGCGAACCCCGGACTGGAGCGACCGGAAACCGGTCCACATGGTGCCTCCAATCAGGAGTCGTCTCTCCCGCTCCATAAGGCTGGCAGGGAAACTCTTCGAGCTGCCTTAGCCGCAGTGCTGCATCATTCTCACTTCTTGGAGGCGTCTTCGTACACGCCTACCTCGAAGTTCTTTCAGGCGCTCGCTTTCATCTTCGTCCCAGCGACAATGAGAGAGTGCATCTCGATCCACATTGGCCAGGCCGGTATCCAGGTCGGAAACGCGTGCTGGGAGCTGTACTGTCTCGAGCATGGCATTCAGCCCGATGGCCAGATGCCGAGTGATAAGACGGTCGGGGGAGGAGACGATGCTTTCAACACGTTCTTCTCGGAGACCGGAGCCGGGAAACACGTTCCCCGCGCCGTGTTCCTCGATCTGGAGCCGACTGTGRTCGATGAGGTGCGGACTGGCACGTACCGTCAGCTGTTTCACCCCGAGCAGCTGATCAGCGGCAAGGAGGATGCCGCCAACAACTTCGCCCGCGGTCACTACACCATCGGGAAGGAGATCGTGGACCTKTGCCTGGACCGCATCCGCAAGCTGGCTGACAACTGCACGGGTCTGCAGGGCTTCCTCGTCTTCAACGCAGTGGGAGGAGGCACCGGGTCCGGTCTGGGCAGTCTGCTCCTGGAGCGTCTTTCTGTGGACTACGGAAAGAAGTCGAAGCTCGGTTTCACCGTGTACCCGTCCCCGCAGGTGTCCACCTCTGTGGTGGAGCCTTACAACAGCGTGTTGTCTACTCACTCTTTGCTGGAGCACACCGATGTGGCCGTCCTCCTCGACAACGAGGCGATCTACGACATCTGCCGGCGTTCGTTGGACATCGAGCGTCCCACGTACACCAACCTGAACAGGCTGGTGTCTCAGGTCATTTCTTCTTTGACCGCTTCTCTCCGTTTCGACGGTGCGCTGAACGTGGACGTGACGGAGTTCCAGACGAACTTGGTGCCATACCCGAGGATCCACTTCATGCTTTCGTCTTACGCTCCCGTGATCTCTGCGGAGAAGGCTTACCACGAGCAGCTGTCGGTTGCAGAGATCACGAACTCTGCCTTCGAGCCTTCTTCTATGATGGCGAAGTGCGACCCCCGCCACGGGAAGTACATGGCTTGCTGCCTGATGTACCGCGGAGATGTGGTCCCCAAGGACGTGAACGCCGCGGTGGCCACCATCAAGACCAAGAGGACCATCCAGTTCGTGGATTGGTGCCCKACCGGGTTCAAGTGCGGCATCAACTACCAGCCGCCGACMGTCGTCCCTGGAGGCGACCTCGCCAAGGTCCAGAGGGCCGTGTGCATGATCTCGAACAGCACCAGCGTGGCGGAGGTGTTCTCCCGCATTGACCACAAGTTCGATCTGATGTACGCCAAGCGCGCTTTCGTGCACTGGTACGTTGGTGAGGGCATGGAGGAGGGAGAGTTTTCGGAGGCCCGTGAGGATCTTGCGGCTCTGGAGAAGGACTACGAAGAAGTGGGTGCCGAGTCCGCGGAGGATGGCGGCGAGGACGATGGTAGCTCTACAGTTCGCATGTTGACATTGGGCCCGAAGACAGTCGGCAGGCAGCAGTCTTGCCCGCCTTGCTGTCTTCGACCGCGCGATGTGAGGGTCGTCAGTAATCTTCACGATGAAGTGCAATCCTGCTCGATTACAGCTCCCAGCATTCGCCTTGAGTGCGGCAATTGTGATTCAGAAAGTCGCTCCCTGCGAACACCTCATCTGAAGTTTAGTGTCGGCATCGAATAAGCCACTACCGGTTGCAAAGAGTGCGCCGTTATAACATTATTCACCAGTGCACATGGCCATTCTGWGTCGGTTTTGGCGAGTCGGCATTGGTTTTGTAACTTACGTGATGATGGGTGTGAGGGTTTGAATCCTGAGACCATGATGCTGAGCTTCCGCAATTCTCACTCTCTAGGCACCAATGCGGTGAACGTTTCGGAAGATGGAGATATGATCGATGTTGTACTGTATAGTACATAGAGCAATCTGTAAGGAACAGTAAAACCCTACACACGCAAAAATGTCTTGAAACAAGGTCTCATGCGATTTACAAGAAGTTTGAAGATTTGTGGTTGTTGGATTGTGTGCAACGAATAGAACGAAGACTCTTGG
